# A crosstalk between the immune system and obstructive sleep apnea

**DOI:** 10.3389/fimmu.2026.1963127

**Published:** 2026-10-07

**Authors:** Sarfraz Ahmed, David Gozal, Alip Borthakur, Abdelnaby Khalyfa

**Affiliations:** 1 Department of Biomedical Sciences, Joan C. Edwards School of Medicine, Marshall University, Huntington, WV, United States; 2 Departments of Pediatrics, Dentistry, Oral and Maxillofacial Surgery, and Biomedical Sciences, Joan C. Edwards School of Medicine, Marshall University, Huntington, WV, United States

**Keywords:** adaptive immunity, chemokines, cytokines, humoral immune response, immune dysregulation, immune response, innate immunity, obstructive sleep apnea

## Abstract

Obstructive sleep apnea (OSA) is characterized by recurrent upper airway obstruction during sleep. Its hallmark pathophysiological features, intermittent hypoxia (IH) and sleep fragmentation (SF), trigger cellular and molecular responses that disrupt immune homeostasis. Recurrent hypoxia-reoxygenation promotes oxidative stress and activates hypoxia-inducible factor-1α (HIF-1α), nuclear factor-κB (NF-κB), and the NLRP3 inflammasome, contributing to sustained inflammatory signaling, endothelial dysfunction, and alterations in circulating and tissue-resident immune cells. This review integrates evidence from clinical and preclinical studies linking OSA to dysregulation of innate, adaptive, and humoral immunity and examines how these immune alterations contribute to OSA-associated systemic morbidity. Innate immune dysregulation in OSA is characterized by activation of neutrophils and monocytes/macrophages, increased oxidative stress, and elevated pro-inflammatory mediators, including IL-1β, IL-6, IL-8, TNF-α, CRP, and MCP-1. Altered expressions of ICAM-1 and VCAM-1 may further promote leukocyte recruitment and endothelial dysfunction, sustaining systemic inflammation and end-organ injury. Adaptive immune dysregulation includes alterations in CD4^+^ and CD8^+^ T-cell populations, Th1/Th17 polarization, disruption of the Th17/Treg balance, and impaired regulatory T-cell function. Emerging, although comparatively limited, evidence also implicates humoral immune dysregulation. Collectively, the interactions among IH, SF, oxidative stress, inflammation, and immune dysfunction support the concept that OSA is a systemic immune-modulating disorder rather than solely an upper airway disorder. Dysregulation of innate, adaptive, and humoral immunity may contribute to OSA-associated metabolic, cardiovascular, and neurocognitive complications. Defining these immune mechanisms may help explain OSA phenotypic heterogeneity and identify biomarkers of disease severity and therapeutic response. Furthermore, characterization of OSA-specific immune signatures may facilitate patient stratification and advance precision-medicine approaches based on individual inflammatory and immunological phenotypes.

## 1 Introduction

Obstructive sleep apnea (OSA) is a chronic major global public health concern. It is one of the most prevalent sleep disorders, affecting nearly one billion adults worldwide (1). OSA is characterized by recurrent episodes of partial or complete upper airway obstruction during sleep. These events result in intermittent hypoxia (IH) and recurrent arousals, which contribute to sleep fragmentation (SF) and heightened sympathetic neural activity (SNA). Population-based studies indicate that 23.4% of women and 49.7% of men suffer moderate-to-severe OSA (2). Nevertheless, the actual prevalence is likely higher because many cases remain undiagnosed, particularly among individuals with mild or nonspecific symptoms. The pathological alterations resulting from upper airway collapsibility contribute to fluctuations and elevations in systemic and pulmonary blood pressures and are associated with an ever-growing number of comorbidities (3–12). The consequences of OSA are far-reaching, ranging from impaired cognitive and physical performance and excessive daytime sleepiness to cardiovascular disease, neuropsychiatric disorders, and carcinogenesis (13–15). A primary consequence of recurrent airflow obstruction is a reduction in arterial oxygen partial pressure, resulting in repeated episodes of inadequate oxygen delivery to tissues and organs followed by reoxygenation. Although each hypoxic episode is transient, their repetitive nature gives rise to a condition known as chronic intermittent hypoxia (CIH) (15–17). CIH is a hallmark feature of OSA and a major driver of systemic inflammation, contributing to the development of cardiometabolic disorders, neurological dysfunction, cellular senescence, carcinogenesis, and immune dysregulation (18). Similarly, disruption of sleep continuity, i.e., SF, as required for termination of the respiratory events elicits a cascade of molecular pathway activations resulting in oxidative stress and inflammation (19).

Sleep–immune interactions represent a fundamental aspect of human physiology. Sleep and circadian rhythms are critical regulators of immune function, exerting profound effects on health and disease susceptibility. The relationship between sleep and immunity is highly dynamic and bidirectional, with disruptions in sleep influencing immune responses and immune activation, in turn, affecting sleep architecture and quality. By modulating inflammatory pathways, neuroendocrine signaling and leukocyte trafficking and activity, sleep and circadian rhythms are essential for maintaining immune homeostasis and coordinating effective immune responses. Consequently, sleep disorders such as OSA can profoundly alter immune function and contribute to systemic inflammation and disease pathogenesis (20). A growing body of evidence highlights a complex bidirectional relationship between OSA and the immune system, with inflammatory mediators playing a critical role in modulating immune cells activation, function, and trafficking. Despite the widespread prevalence of OSA, the immunological mechanisms underlying its systemic consequences and increased disease susceptibility remain incompletely characterized. Recent studies have shown that OSA is variably yet frequently associated with elevated levels of pro-inflammatory cytokines and other inflammatory biomarkers, suggesting that chronic inflammation is a key contributor to the development of its cardiovascular, metabolic, neurological, and immune-related complications (21, 22). These observations highlight the need for a deeper understanding of the inflammatory mechanisms underlying OSA and their impact on immune system function. Mediators of the immune system, especially cytokines such as IL-1 (interleukin-1), IL-6, TNF-α (tumor necrosis factor), PGD2 (Prostaglandin D2) and many others are involved in the regulation of physiological sleep (23–27). Through activation of the immune system and the release of sleep regulatory cytokines such as TNF-α, cytokines and several other inflammatory markers, the muramyl peptides and gut microbial products have been shown to contribute to the homeostatic regulation of sleep. Together with current advances, the research suggests a pivotal role of sleep and immune system, and thus, the occurrence of alterations in the immune system by sleep disorders such as OSA (22, 28–30). It has also been reported that IH in patients with severe OSA interferes with the innate and adaptive immune response (22, 31, 32), promotes cellular angiogenesis and lymphatic angiogenesis (33–35). The present narrative review aims to outline the established research progress regarding cellular innate, adaptive and humoral immune system in the context of OSA. We primarily emphasize evidence from *in vivo* and clinical studies investigating OSA, its immunopathology and alterations in immune system components, including interactions within the gut–brain–immune axis. While recent research has largely concentrated on the prevalence of sleep disturbances and sleep–wake dynamics, investigations specifically addressing the immune mechanisms and immunopathological consequences of OSA remain comparatively scarce.

OSA exerts pathological effects across multiple organ systems through systemic inflammation, with IH and SF serving as the principal upstream drivers (19, 36). Cyclic hypoxia generates reactive oxygen species (ROS) that activate redox-sensitive transcription factors, particularly nuclear factor kappa B (NF-κB), triggering downstream inflammatory cascades (19, 37). This response preferentially activates monocytes and macrophages, while upregulation of Toll-like receptors (TLR2 and TLR4) on circulating monocytes amplifies innate immune signaling (19, 38) and the release of pro-inflammatory mediators including TNF-α, IL-6, IL-8 and others (19, 38). This inflammatory milieu promotes endothelial dysfunction, reduces nitric oxide bioavailability and sustains immune activation. These changes contribute to neurological complications, including stroke and cognitive dysfunction (39), cardiovascular disease, including hypertension, coronary artery disease, myocardial infarction, arrhythmia and heart failure (19, 40, 41), and metabolic dysfunction, including insulin resistance and type 2 diabetes mellitus (41, 42). Chronic IH also promotes hepatic injury through lipid dysregulation, oxidative stress, and HIF-1α–mediated inflammation, contributing to the development and progression of metabolic dysfunction-associated steatotic liver disease (MASLD) and, in more advanced cases, metabolic dysfunction-associated steatohepatitis (MASH) (43), activating NLRP3 inflammasome which further enhances IL-1β and IL-18–mediated tissue injury (41, 42). In summary, systemic inflammation represents a common pathway through which OSA-related IH and SF exacerbate multiorgan morbidity (44). **Figure 1** illustrates the systemic impact of obstructive sleep apnea (OSA) on multiple organ systems. OSA-induced IH, sleep fragmentation and sympathetic activation contribute to dysfunction in various organs, including cardiovascular, metabolic, neurological, and respiratory systems.

**Figure 1:**
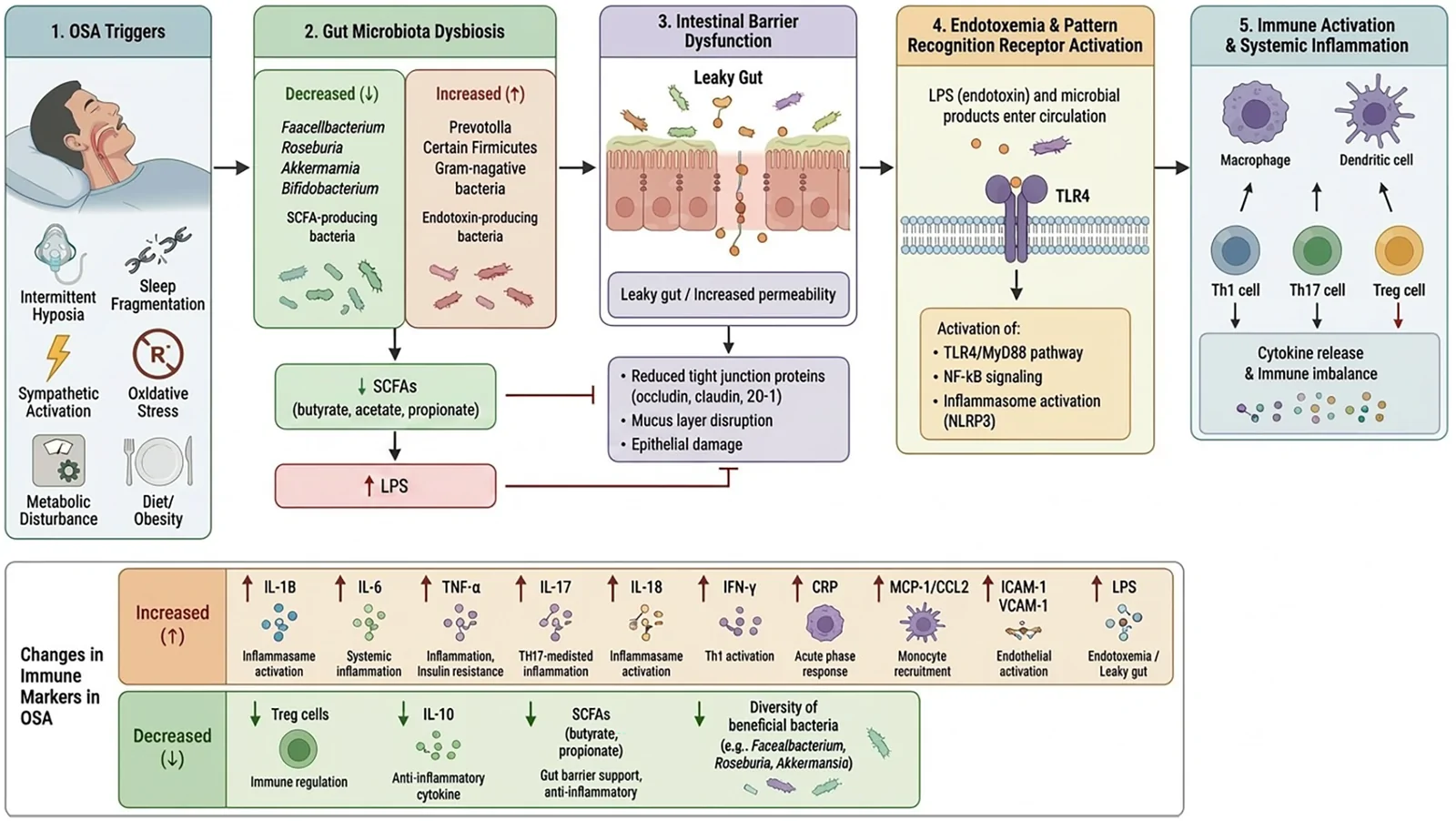
Schematic illustration of the proposed role of systemic inflammation as a common pathway linking obstructive sleep apnea (OSA) to multisystem disease. OSA affects multiple organs, including the brain, heart, lungs, liver, kidneys and metabolic tissues, leading to increased macrophage activation, pro-inflammatory cytokine production, oxidative stress, and immune activation. These inflammatory processes contribute to neurological, cardiovascular, pulmonary, renal, hepatic and metabolic morbidities. OSA, Obstructive Sleep Apnea; IL-6, Interleukin-6; TNF-α, Tumor necrosis factor-alpha; ESRD, End-stage Renal-Disease; MAFLD, Metabolic Dysfunction-associated Fatty liver Disease; MASH, Metabolic Dysfunction-associated Steatohepatitis.

### 1.1 Literature search methodology

This review was conducted as a non-systematic narrative review and did not follow a formal systematic review protocol or include a meta-analysis. Relevant literature was identified through searches of Google Scholar, PubMed, Scopus, Embase, and Web of Science using combinations of keywords, including “obstructive sleep apnea,” “OSA,” “immune system,” “immune dysregulation,” “inflammation,” “intermittent hypoxia,” “sleep fragmentation,” “sleep deprivation,” “innate immunity,” “adaptive immunity,” “humoral immunity,” “cytokines,” “chemokines,” “immune cells,” and “gut–brain axis.” Emphasis was placed on recent advances and key studies addressing the bidirectional interactions between OSA and immune regulation. Relevant experimental and clinical studies examining intermittent hypoxia, sleep fragmentation, and, where mechanistically appropriate, sleep deprivation or restriction were also included.

## 2 Sleep, OSA and immune directionality

As outlined in the introduction section, sleep and circadian rhythms regulate immune-cell trafficking, cytokine expression, adaptive immune responses and immunological memory (28, 45–48). The changes in sleep are likely to have a nexus with disease pathogenesis and immune dysregulation (49). Extant literature also reveals a similar association between OSA and altered immune response (22, 29, 33–35). Current findings reinforce the bidirectional relationship between sleep, OSA, inflammation and immune regulation, highlighting a closely interconnected pathway through which sleep disruption may contribute to immune-mediated disease mechanisms and vice-versa. In OSA, recurrent IH and SF are associated with systemic inflammation, altered leukocyte profiles and dysregulated cytokine production, with reported differences according to disease severity and sex (50–53). However, the independent contributions of IH and SF are difficult to distinguish because these disturbances occur concurrently in clinical OSA. Obesity is assumed a potential confounding factor when interpreting immune dysregulation associated with OSA (54–56). It has been reported that obesity frequently coexists with OSA (54, 56). Thus, it may contribute to systemic inflammation and immune dysregulation including changes in circulating leukocytes and pro-inflammatory cytokines (54–56). Therefore, some immune perturbations reported in patients with OSA may be partly attributable to obesity rather than OSA itself. Studies using BMI-matched controls or adjusting for BMI and other metabolic variables are particularly important for distinguishing the independent effects of OSA on immune dysregulation. Although experimental sleep deprivation and sleep restriction do not directly model OSA-associated sleep fragmentation, these paradigms provide complementary evidence regarding the inflammatory and immune consequences of disrupted or insufficient sleep and are relevant to understanding the interconnected and bidirectional relationships among sleep, OSA, and immune regulation. These studies are discussed where appropriate to provide additional mechanistic context.

## 3 Interplay between OSA and the immune system

Numerous experimental studies have probed the effect of experimental sleep manipulation on multiple immune markers. These markers encompass number of leukocytes, leukocyte subsets in various tissues and blood circulating cytokines, cytokines production in leukocytes, blood antibodies and complement factors, as well as functional aspects such as cell cytotoxicity (28, 33, 48, 57–61). Evidence from experimental and clinical studies indicates that sleep disturbances are associated with dysregulation of immune cell populations, inflammatory markers and indicators of cellular aging. These immune alterations may represent a potential mechanism through which impaired sleep contributes to increased disease risk, as demonstrated in recent studies. However, the mechanisms underlying the effects of sleep and OSA on immune function remain incompletely understood. Current evidence supports the hypothesis that sleep promotes an immunologically supportive environment by facilitating the migration of antigen-presenting cells (APCs) and T cells to lymphoid tissues and enhancing their interactions with other immune cells, thereby supporting the initiation and coordination of adaptive immune responses (28, 33, 48, 57–61). These processes may promote more robust adaptive immune responses and the development of long-lasting immunological memory. In OSA, however, such mechanisms may be impaired or even reversed, potentially contributing to immune dysregulation. Accordingly, numerous studies have investigated the complex interactions between OSA and the immune system (39, 62–69). The effects of OSA on the functional aspects of innate, adaptive and humoral immunity have been less extensively investigated. Comparisons across studies are often challenging due to methodological heterogeneity, which may contribute to inconsistencies in reported findings. The following sections examine the complex interplay between OSA and the innate, adaptive and humoral immune systems, with particular emphasis on immune-inflammatory responses, cytokine and chemokine signaling, and the bidirectional interactions among the immune system, gut microbiota and the brain.

## 4 Innate and adaptive immune responses in OSA

### 4.1 Leukocytes and leukocyte subsets

The immune system serves as the body’s primary defense mechanism, recognizing and eliminating both endogenous and exogenous threats. Its effector cells, known as leukocytes or white blood cells (WBCs), are widely distributed throughout the body. These cells are classified based on their developmental origin, structural characteristics, surface markers, functional properties and degree of granularity. Leukocytes are broadly categorized into two major groups: 1) granulocytes, which are predominantly cells of the innate immune system and include neutrophils, eosinophils and basophils, while mast cells arise from related myeloid progenitors and reside in tissues (70); and 2) agranulocytes, which include monocytes, natural killer (NK) cells, dendritic cells (DCs) and lymphocytes. Monocytes can differentiate into macrophages and DCs, whereas lymphocytes comprise B lymphocytes or B cells (which mature in the bone marrow) and T lymphocytes or T cells (which mature in the thymus) that are central components of the adaptive immune system. These cells also express specialized receptors, including B-cell receptors (BCRs) and T-cell receptors (TCRs), which recognize specific antigenic peptides (28, 61, 71). Leukocytes stem, evolve and interact primarily in bone marrow and thymus, and secondarily in spleen, lymph nodes and mucosa associated lymphatic tissue. The traffic works among these tissues and lymphoid organs throughout the body via the rout of lymphatic vessels and bloodstream. They interact with each other and other non-immune cells through soluble factors like cytokines, chemokines, surface markers and vesicles. Their prime defense mechanisms can be categorized into humoral immunity comprising soluble mediators and cellular immunity comprising cytotoxicity and phagocytosis (28, 61, 71).

Total leukocyte counts and leukocyte subsets represent some of the most extensively investigated immunological parameters in sleep research. Current evidence indicates that sleep has a modest yet significant effect on circulating leukocyte populations. Several human studies have demonstrated a reduction in total leukocyte counts following sleep deprivation (72–75). Some studies reported no such reduction in leukocytes (76–80). Notably, none of the studies reported an increase in leukocyte counts following sleep disruption. Collectively, the available evidence suggests that sleep deprivation is generally associated with a reduction in circulating leukocyte numbers. Acute sleep deprivation has been shown to decrease total monocyte and lymphocyte counts, as well as several lymphocyte subsets (76, 81–83) or unchanged (75, 84, 85). In OSA, multiple studies have reported a similar pattern of increase, decrease or unchanged levels of leukocytes, lymphocytes and their subsets (28, 33, 57–61). These variations reveal a homeostatic response. A detailed review of OSA-specific studies investigating leukocytes, lymphocytes, and their respective immune-cell subsets will be presented in the following sections.

#### 4.1.1 Neutrophils, eosinophils and basophils

Pertaining to simple sleep deprivation, research has shown no changes in the granulocyte subsets of basophils and eosinophils (75, 84, 85). Conversely, few studies show that sleep deficiency reduces neutrophils count (72–75, 86), while some data revealed no changes in neutrophils numbers (78, 84). However, based on the limited existing studies, the primary focus of granulocytes in OSA patients was on neutrophils numbers. The results of a cross-sectional OSA study comprising 1,298 adults showed that the patients with severe OSA (Apnea-hypopnea index (AHI) ≥30/hr) exhibit significantly a higher count of neutrophils when compared to the patients with less severe OSA, with no significant changes in eosinophils or basophils counts. This study suggests that OSA is independently associated with a higher neutrophils count, suggesting the presence of inflammatory responses (87). Another retrospective observational study examining 277 individuals revealed that OSA patients had an elevated level of basophils compared to non-OSA healthy participants. The study disclosed that OSA was associated with a systemic immune response, notably involving basophils (88). Hoffmann et al. investigated the effects of reactive oxygen species (ROS) production in granulocytes from OSA patients compared to healthy controls (89). The granulocytes were exposed to hypoxic conditions and quantification of ROS levels was performed. ROS production was increased under hypoxia in both groups; however, a more significant increase was observed in patients with OSA (89). However, the study was performed *in vitro* and lacks detailed information on the sample size, methodologies and with no quantification of granulocytes, which are critical for assessing the results’ robustness. An investigation of blood samples from OSA patients demonstrated a reduced ability of neutrophils to phagocytose bacteria, along with decreased NADPH oxidase activity (60), indicating significant impairment of neutrophil function. Multi-center research investigated the neutrophil-to-lymphocyte ratio (NLR) as a biomarker of inflammation in OSA patients Interestingly, none of the studies reported an increase in leukocyte counts following sleep disruption. Finally, work by Lena Lavie and colleagues has clearly shown the reduced apoptosis observed in neutrophils and sought to identify specific mechanisms (90–92). Taken together, the available evidence suggests that sleep deprivation is generally associated with a reduction in circulating leukocyte numbers. Specifically, acute sleep deprivation has been shown to decrease total monocyte and lymphocyte counts, as well as several lymphocyte subsets. NLR was found to be significantly higher in patients with severe OSA (AHI ≥30/hr) compared to the patients with moderate or mild OSA and as well as healthy controls. Intriguingly, levels of C-reactive protein (CRP) did not show substantial variations between these groups, demonstrating that NLR may be a vital and a more sensitive marker of inflammation in OSA (93). Several other studies have reported an increased NLR in OSA patients, suggesting its role as a marker of inflammation (53, 94, 95). Across all these studies, neutrophils elevation consistently emerged as a key finding in the patients with severe OSA, particularly suggesting it to be a pro-inflammatory marker. The NLR was also corroborated to be a useful diagnostic marker for OSA severity. While these studies highlighted the inflammatory role of neutrophils and NLR associated with OSA, however these studies failed to probe the role of other immune cells in OSA pathology.

Experimental studies further support the role of SF in neutrophil-mediated inflammation. An increased of neutrophil populations was increased in the blood, bone marrow, spleen, aorta and small intestine following chronic SF, consistent with SF-induced myelopoiesis and systemic inflammation (96). Similarly, SF increased neutrophil progenitor populations within the bone marrow, indicating enhanced granulopoiesis and activation of innate immune pathways (97). Using the same Apoe^−^/^−^ mouse model, another study demonstrated that SF induced neutrophilia through increased neutrophil production and mobilization from the bone marrow, thereby contributing to systemic inflammation. Chronic SF increased neutrophil abundance in bone marrow, blood, spleen and small intestine. Importantly, SF increased the proportion of neutrophils within carotid artery plaques, where their accumulation was associated with larger necrotic cores, extracellular matrix disorganization and reduced collagen content, suggesting impaired plaque stability (98).

Collectively, neutrophil elevation consistently emerges as a characteristic finding in severe OSA and supports a role for neutrophils as contributors to OSA-associated inflammation. Furthermore, NLR has been proposed as a useful biomarker of disease severity. Although these studies highlight the inflammatory significance of neutrophils and NLR in OSA, most have not comprehensively evaluated the contribution of other immune cell populations to OSA pathophysiology.

#### 4.1.2 Monocytes

Redistribution of monocyte subsets has been explored as an approach aimed to distinguish between inflammatory alterations specifically associated with OSA and those related to obesity, since both of these conditions are linked to systemic inflammation (54). Various studies have shown that sleep deprivation elicits a decrease in number of total monocytes count (76, 81, 83, 99). A recent investigation examined the impact of OSA (AHI: 17.8 ± 17.0 events/hr) on various subtypes of monocytes before and after positive airway pressure (PAP) therapy. The monocyte subtypes included classical monocytes such as CD14++CD16−, non-classical monocytes like CD14dim+ CD16+ and intermediate monocytes such as CD14+ CD16 +. Compared with healthy subjects, untreated OSA patients exhibited an increase of intermediate monocytes and a decrease of classical monocytes, as determined by flow cytometric analysis (100). Following PAP therapy, intermediate monocytes (CD14++CD16+) decreased whereas classical monocytes increased toward control levels (100). Notably, monocyte subset distribution in the PAP-treated cohort was not significantly associated with BMI, although most participants were overweight or obese, limiting complete separation of the effects of OSA from adiposity (100). In contrast, these changes were positively correlated with body mass index (BMI) in an earlier study, with obese patients exhibiting the highest frequencies of CD16+ monocytes (101). In addition, a substantial increase in complex formation between CD14+ monocytes and CD3+ T cells was observed, which has been reported as a potential marker of immune dysfunction (101, 102). Thus, obesity may contribute to OSA-associated monocyte perturbation and the independent effect of OSA remains uncertain in studies without BMI-matched controls or adequate adjustment for adiposity. Another retrospective observational study reported increased monocyte levels that were positively correlated with OSA severity and inflammation (88). Although BMI differed between the groups, monocyte levels were not significantly associated with BMI, whereas AHI remained an independent predictor. The findings suggested that the observed monocyte changes may be related to OSA beyond the effects of obesity (88). Flow cytometric analysis demonstrated a significant reduction in classical monocytes (CD14++CD16−) and an increase in intermediate monocytes (CD14++CD16+) in normal-weight OSA patients, obese subjects without OSA and obese OSA patients compared with controls, indicating dysregulation of monocyte phenotypes (54). Intriguingly, these findings suggest overlapping but distinct effects of OSA and obesity on monocytes dysregulation. CD11b expression was increased in normal weight OSA and obese OSA patients. This supports an OSA-related effect independent of obesity. However, CD11c expression was more significantly increased in obesity. A decreased CD29 expression was observed only in patients with both OSA and obesity, suggesting a possible additive effect of the two conditions. Increased expression of adhesion molecules and chemokine receptors, including CD11a, CD11b, CD11c and CX3CR1, further suggested enhanced immune activation (54). In addition, inflammatory mediators such as lipocalin and matrix metalloproteinase-9 (MMP-9) were significantly elevated. Experimental studies using THP-1 monocytes exposed to hypoxia, leptin and TNF-α also demonstrated enhanced cytokine secretion and adhesion molecule expression, supporting the direct role of OSA- and obesity-related stimuli in modulating monocyte inflammatory responses (54). Finally, the pathophysiologic roles played by CD11 monocytes in aspects of OSA-induced morbidity such as metabolic dysfunction and insulin resistance have been recently delineated in a murine model (103). In addition, the role of nitric oxide production by monocytes in endothelial function of children with OSA further buttresses the concept of a major role for monocytes in OSA-induced morbidities (104).

Moriarty et al. (2022) reported that female ApoE−/− mice fed a high-fat diet and exposed to chronic sleep fragmentation (SF) exhibited increased pro-inflammatory monocyte populations in peripheral blood, bone marrow, spleen, aorta and small intestine, indicating enhanced innate immune activation (96). Furthermore, McAlpine et al. (2019) showed that chronic SF significantly increased circulating and bone marrow Ly-6C^high^ pro-inflammatory monocytes, accompanied by enhanced monocytosis, expansion of monocyte progenitors and increased myelopoiesis in animal models (97). These alterations were associated with accelerated atherosclerotic lesion development, indicating that SF promotes inflammatory monocyte production and mobilization through enhanced bone marrow hematopoiesis (97). Similarly, chronic SF increased circulating pro-inflammatory monocytes and enhanced bone marrow hematopoiesis, further supporting a role for SF in driving innate immune activation (105). Overall, these findings suggest that OSA independently and synergistically alters monocyte subset distribution and activation, highlighting monocyte phenotyping as a potential biomarker of inflammation in OSA (77).

#### 4.1.3 Dendritic cells and macrophages

Antigen-presenting dendritic cells (DCs) are classified into myeloid (mDCs) and plasmacytoid (pDC) dendritic cells. These cells can be further differentiated into subsets such as mDC2 (CD141+) and mDC1 (CD1+). They have diverse functions including stimulation of CD4+ and CD8+ T cells, release of different cytokines and phenotyping of Th1 and Th2 cells. Because of their scant presence in peripheral blood, accurate analysis of DCs is challenging, particularly for subsets. Two pertinent studies examined the impact of OSA on DCs in OSA patients compared with healthy subjects, one reported increased DCs levels (58), while the other one found no changes (106). Conversely, a marked reduction in circulating dendritic cells (DCs) was reported in patients with moderate-to-severe OSA. The other subsets such as mDC1, mDC2, pDC and mDC2 were found to be impaired with the least limit of detectability in some patients (107). The decline in mDC2 and pDC was found to be inversely associated with the concentration of IL-6, known to be an inhibitor of DCs’ functional maturation (107).

Intermittent hypoxia (IH) promotes macrophage recruitment and polarization toward a pro-inflammatory phenotype. For example, in male C57BL/6J mice, IH increased aortic CD11b^+^/F4/80^+^ macrophage accumulation and enhanced Ly6c and CD36 expression while reducing CD64 expression, indicating a shift toward a pro-inflammatory and pro-atherogenic macrophage phenotype (108). Similarly, IH increased M1 macrophage accumulation in epididymal adipose tissue in both lean and obese mice without significantly affecting M2 macrophages. This showed an obesity independent effect of IH on pro-inflammatory M1 polarization (109). In contrast, obese mice exhibited lower M2 macrophage levels than lean mice, indicating an additional obesity-associated effect on macrophage phenotype (109). Chronic SF increased F4/80^+^ macrophage accumulation and foam cell formation in mice and was associated with insulin resistance, oxidative stress and enhanced M1 macrophage polarization in visceral adipose tissue (110, 111). In high-fat diet–fed Apoe^−^/^−^ mice, SF further increased circulating myeloid cells, elevated brain CD11b expression and enhanced CD68^+^ macrophage accumulation within aortic lesions, indicating systemic myeloid activation, neuroinflammation and accelerated atherosclerotic progression (105). Both SF and control groups were maintained on a high-fat diet, supporting an additional effect of SF under obesity-prone metabolic conditions. However, the absence of a lean-diet control limits the ability to determine whether these effects are independent of obesity. Additional evidence supporting a causal role for macrophages in IH-induced inflammation was provided by CD11b–DTR transgenic mice, in which selective depletion of CD11b^+^ cells markedly reduced macrophage infiltration in visceral white adipose tissue and liver following IH exposure, demonstrating a direct contribution of myeloid cells to tissue inflammation (103).

#### 4.1.4 Natural killer and natural killer T cells

Natural killer (NK) cells and NK T cells are an important component of the innate immune system and play a major role in the early immune response. These cells represent a significant lymphocyte population with immunoregulatory properties and are thought to be dysregulated in OSA (60). Alterations in NK cells may contribute to systemic inflammation and the metabolic and cardiovascular comorbidities associated with OSA and obesity (112). NK cells are shown to be increased in number when resuming or increasing normal sleep intensity (113, 114). In contrast, acute sleep deprivation has been observed to lead to a reduced number of NK cells (76, 81, 83, 99). However, some reports suggest an increased number of lymphocyte subsets of NK cells in sleep deprivation (73, 80, 115). Only a few studies showcasing shifts of natural NK cells and NKT cells are available in OSA patients. For example, a study comprising 48 OSA patients, revealed that the percentage of NK and NKT cells was elevated in OSA patients compared to healthy control subjects (58). NKT-like cell levels were higher in obese patients with OSA than in non-obese patients with OSA, suggesting that obesity may further enhance OSA-associated immune activation. However, their association with the oxygen desaturation index also suggests a contribution of OSA-related hypoxemia. Notably, NKT cells are actively involved in immunological processes linked with atherosclerotic alterations (116). Conversely, decreases in NK and NKT cells such as CD3+/CD16+ CD56+; CD3−/CD16+ CD56+ in OSA patients along with a positive correlation of this decrease with the nadir oxygen saturation have also been described (106, 117). Another recent investigation of OSA in children also revealed a decreased percentage of NK cells following restoration of normal sleep breathing patterns through surgical intervention (118). Overall, findings regarding NK and NKT cells in OSA remain inconsistent. These discrepancies may reflect differences in study populations, including age, obesity, OSA severity, and the degree of nocturnal hypoxemia, as well as differences between experimental sleep deprivation paradigms and clinical OSA (58, 73, 76, 80, 81, 83, 99, 106, 113–115, 118). Furthermore, variations in blood-sampling time, NK/NKT cell phenotyping, and associated comorbidities may contribute to the inconsistent findings. Thus, current evidence supports dysregulation of NK and NKT cells in OSA, although the magnitude and direction of these changes remain heterogeneous across studies.

#### 4.1.5 B cells

The effect of normal sleep on leukocyte distribution has been associated with an increase in B-cell numbers within the murine spleen (113, 114). Studies have shown that casual sleep deprivation led to a reduced number of total lymphocytes and the lymphocytes subsets such as B cells (76, 81, 83, 99) or alternatively resulted in no changes (72, 74, 77, 78). B cells act as a humoral defense system and secrete several cytokines and their cofactors such as IL-10, PD-L1, which have been associated with the induction of T cells death or apoptosis. A decrease in B cells has been shown in severe OSA patients, with a vigorous association to obesity (58, 119). Conversely, it has been shown that an increase in a subset of B cells like CD19+ B lymphocytes occurs in patients with moderate to severe OSA (106). This discrepancy is likely because the patients recruited in these contradictory studies differed in the presence of comorbidities, while the OSA patients in the Domagala-Kulawik’s investigation had several comorbidities which may influence the differentiation and expression of immune cells like B lymphocytes. Evidence regarding B-cell responses to sleep disruption *in vivo* models remains limited. In a murine model of acute sleep deprivation, mice exposed to 6 hours of sleep deprivation and characterized immune cell populations within the brain parenchyma, meninges, and choroid plexus using high-dimensional mass cytometry (CyTOF) (120). Sleep deprivation significantly increased B-cell abundance across these CNS compartments, suggesting enhanced recruitment or retention of B cells within the central nervous system (120). Similarly, in a mouse model of IH, thoracic aortic immune responses were evaluated using RNA sequencing, immune cell deconvolution analyses and immunohistochemical techniques (121). These analyses demonstrated increased B-cell infiltration within peri-aortic tissues, indicating a potential role for B cells in IH-induced vascular inflammation and remodeling (121).

#### 4.1.6 T cells

Sleep fragmentation has been reported to reduce total lymphocyte counts and lymphocyte subsets, including CD4^+^ and CD8^+^ T cells (76, 81, 83, 99), although some studies observed no significant changes in these cell populations (72, 74, 77, 78). In contrast, other investigations demonstrated increased numbers of CD4^+^ and CD8^+^ T cells following sleep deprivation (73, 80, 115). Several studies have further examined distinct T-cell subpopulations in OSA. It has been reported that reduced levels of CD4^+^CD25^+^Foxp3^+^ regulatory T (Treg) cells are present in the peripheral blood of OSA patients, which positively correlated with disease severity (122). In contrast, when Treg cells were assessed using CD4^+^CD25^+^/CD127^+^ markers, no significant differences in Treg frequencies were observed between 39 patients with OSA and 33 healthy controls (106). Activated Treg cells are known to suppress established immune responses through secretion of anti-inflammatory cytokines such as IL-10, leading to downregulation of effector T cells (123). γδ T cells have also been implicated in OSA-associated immune dysregulation. A study demonstrated that γδ T cells isolated from OSA patients exhibited a 2.5-fold increase in cytotoxicity against endothelial cells, suggesting that hypoxia-induced lymphocyte dysfunction may contribute to endothelial injury in OSA. Additionally, the proportion of CD3^+^/γδ T cells was reported to be reduced in obese OSA patients compared with healthy subjects (123). A similar reduction was observed in non-obese OSA patients, although it did not reach statistical significance, while sleep-related parameters remained associated with these cells after accounting for BMI and other confounding variables, supporting an OSA-related effect beyond obesity. γδ T cells express T-cell receptors composed of γ- and δ-subunits, which participate in specialized immunological signaling pathways that remain incompletely understood. Patients with mild-to-moderate OSA have also demonstrated increased CD4^+^ helper T (Th) cells, accompanied by reductions in central and effector memory T-cell populations, monocytes and associated cytokines, alterations that may contribute to chronic inflammatory disease (101). Conversely, a decreased CD4^+^/CD8^+^ T-cell ratio was observed in patients with severe OSA (58). Furthermore, increased proportions of CD8^+^ T cells, particularly CD8^+^CD56^+^perforin^+^ subsets, have been reported, suggesting a potential role for these cells in mediating inflammatory responses (112). Similarly, increased proliferative activity of CD4^+^ T cells accompanied by neutrophil dysfunction has been reported, suggesting altered cellular immune responses in patients with OSA (60). In contrast, reduced concentrations of cytotoxic CD3^+^CD8^+^ T cells have been observed in patients with severe OSA, with cell counts negatively correlating with the lowest oxygen saturation levels. Consequently, the CD4^+^/CD8^+^ ratio was positively correlated with the apnea-hypopnea index (AHI) and inversely associated with oxygen saturation (106). A recent interventional study involving 36 children with OSA, 32 children with tonsillar hypertrophy but not clinically present OSA and 30 healthy controls demonstrated decreased percentages of CD3^+^ and CD4^+^ T cells, along with elevated percentages of CD8^+^ T cells and an altered CD4^+^/CD8^+^ ratio. Surgical intervention resulted in significant improvements in these immune markers at 3 to 6 months follow-up (118).

Animal studies further support the effects of sleep disruption on T-cell responses. In an experimental autoimmune encephalomyelitis (EAE) model, sleep fragmentation significantly increased both the number and proportion of CD4^+^ T cells infiltrating the spinal cord, indicating enhanced CNS-directed T-cell trafficking during active neuroinflammation (124). In contrast, short-term sleep deprivation in female C57BL/6J mice reduced splenic expression of genes involved in immune cell recruitment and antigen presentation without significantly affecting T-cell proliferation, SRBC-specific antibody production, or TCR repertoire diversity, suggesting molecular alterations in adaptive immunity without overt impairments of primary immune responses (125). Another study demonstrated that 4 weeks of IH exposures in male mice led to a reduction in splenic CD4^+^CD25^+^Foxp3^+^ regulatory T-cell (Treg) levels, indicative of a shift toward a pro-inflammatory Th17/Treg imbalance. After 4 weeks of normoxic recovery, Treg levels and T-cell subset distributions normalized, suggesting that IH-induced immune dysregulation may be reversible following the resolution of hypoxic stress (126).

##### 4.1.6.1 Th1/Th2 immune imbalance

Th1 and Th2 cells are functionally distinct CD4^+^ T-cell subsets that regulate cellular and humoral immunity, respectively. Sleep fragmentation and chronic IH alter adaptive immune responses by disrupting the balance between Th1 and Th2 cytokine activity. Experimental sleep deprivation studies have demonstrated shifts in the Th1/Th2 cytokine ratio, characterized by suppression of IL-2 and a relative enhancement of Th2-associated immune responses following prolonged sleep restriction (82, 127). In OSA, such alterations may contribute to persistent systemic inflammation, impaired cellular immunity and increased susceptibility to metabolic and cardiovascular complications.

##### 4.1.6.2 Th17/Treg dysregulation

Th17 cells are a pro-inflammatory subset of CD4^+^ T cells that also play important roles in neutrophil recruitment and inflammatory responses. Increasing evidence suggests that OSA is associated with a disruption of the Th17/Treg balance. Studies have reported elevated frequencies of Th17 cells, increased circulating levels of IL-17 and IL-6, enhanced expression of the Th17-associated transcription factor RORγt and increased Th17/Treg ratios in patients with OSA, whereas Treg frequencies and the expression of Foxp3 and TGF-β1 are reduced (29, 122, 126). In a study involving 44 male OSAS patients and 20 healthy controls, increased Th17/Treg ratios were positively correlated with high-sensitivity C-reactive protein levels and disease severity as assessed by the apnea–hypopnea index, supporting a link between Th17/Treg imbalance and systemic inflammation in OSA (122). These findings indicate impaired immunoregulatory function and enhanced inflammatory activity that may contribute to OSA pathogenesis and its associated comorbidities. Notably, positive airway pressure (PAP) therapy has been shown to partially reverse these abnormalities. In contrast, Polasky et al. reported no significant changes in Th1 or Th17 cell populations, highlighting variability among studies and the need for further investigation (101).

Collectively, these studies demonstrate significant alterations in T-cell subsets, including T-helper (Th) cells, and broader leukocyte immune responses in OSA, although findings are not always consistent across studies. This heterogeneity may reflect differences in patient characteristics, including oxygen desaturation, obesity, age, and disease severity, as well as experimental conditions and immunophenotyping approaches. Differences in the markers used to define Tregs and other T-cell subsets may further contribute to reports of increased, decreased, or unchanged cell populations. In addition, most studies have focused on peripheral blood immune cells, with relatively limited investigation of animal models, lymphoid tissues, or tissue-resident immune populations. Methodological differences in sampling time and site, marker selection, and therapeutic interventions may also contribute to variability across studies. Overall, the available evidence supports dysregulation of T-cell and broader leukocyte immune responses in OSA rather than a uniform directional change across immune cell subsets. However, the mechanisms underlying these alterations, including the tissue distribution and redistribution of specific immune cell populations, remain incompletely understood.

## 5 Humoral immunity in OSA

Research on the effects of sleep disturbance and OSA on humoral immunity, including immunoglobulins and complement proteins, remains limited. Importantly, findings from experimental sleep-deprivation studies in humans or animal models should be distinguished from direct evidence in patients with OSA. Although sleep-deprivation models may reproduce selected features relevant to OSA, they do not capture key disease-specific characteristics, including recurrent upper-airway obstruction, intermittent hypoxia (IH), and associated physiological disturbances and comorbidities. Moreover, direct evidence from OSA populations is sparse and is derived largely from pediatric studies, with relatively few adult cohorts. Thus, current evidence regarding humoral immune alterations in OSA remains preliminary and should be interpreted with caution.

### 5.1 Immunoglobulins (IgA, IgG and IgM)

#### 5.1.1 Evidence from experimental sleep deprivation models

In adults, a single night of total sleep deprivation has been associated with increased circulating IgG, IgM and IgA levels the following morning. These changes may reflect alterations in antibody binding or consumption rather than *de novo* synthesis (128). Circulating IgA decreased after four days of selective REM-sleep restriction, whereas IgG and IgM remained unchanged in healthy male individuals (129). Another study found increased salivary secretory IgA levels following one and two days of sleep deprivation in male participants; however, this increase may have resulted from antibody accumulation secondary to reduced salivary flow rather than increased production (79). These studies involve small samples of healthy subjects exposed to acute experimental sleep loss; hence their findings cannot be directly extrapolated to patients with OSA.

In contrast to reports of impaired humoral immunity following sleep disruption, Renegar et al. (1998) demonstrated that repeated sleep deprivation in influenza-immunized mice did not diminish serum antibody levels. Rather, influenza-specific IgG concentrations were transiently increased, possibly reflecting altered antibody catabolism rather than enhanced antibody synthesis (130). A subsequent study in young and aged mice similarly demonstrated that short-term sleep deprivation did not suppress serum IgG responses or abolish secretory IgA-mediated mucosal protection in the upper respiratory tract (131). Other animal studies suggest that chronic sleep disruption may adversely affect humoral immunity. In mice, sleep deprivation reduced IgG2b class switching through circadian clock-dependent alteration of CD4^+^ T-cell TGF-β1 signaling (132). Likewise, chronic sleep fragmentation impaired influenza-vaccine responses in mice, resulting in lower hemagglutination-inhibition and neutralizing antibody titers, reduced vaccine-specific IgG responses, and defective germinal-center and plasma-cell differentiation (133). Chronic sleep restriction also increased serum IgM in rats without affecting IgG or IgA (134). These findings suggest that the humoral consequences of sleep loss depend on its duration, type and the immune outcome examined. Nevertheless, these animal models provide mechanistic hypotheses only and do not constitute direct evidence of equivalent humoral dysfunction in human OSA.

#### 5.1.2 Patients with OSA

Direct evidence concerning immunoglobulins in OSA remains sparse. In a retrospective cross-sectional study of 110 children with adenotonsillar hypertrophy, elevated serum IgA was independently associated with moderate-to-severe OSA, whereas IgM was not independently associated with disease severity (135). Because this study involves a selected pediatric population with adenotonsillar hypertrophy, its outcomes cannot be generalized to adults OSA phenotypes. Moreover, its cross-sectional design establishes association rather than causation. Much of the discussion concerning mucosal IgA in OSA is otherwise based on indirect evidence from related upper-airway inflammatory disorders, including allergic rhinitis and asthma (136). Secretory IgA (SIgA) serves as an important mucosal defense mechanism in the upper respiratory tract and disruption of SIgA function may contribute to persistent mucosal inflammation. Considering the altered tonsillar microbiota observed in pediatric OSA, SIgA dysfunction could promote epithelial-barrier disruption and chronic upper-airway inflammation. However, this proposed mechanism has not been directly evinced in OSA and should therefore be regarded as a hypothesis rather than an established feature of the disease.

A study by Sarmiento Varón and colleagues provided evidence of local immune dysregulation in hypertrophied tonsils from children with OSA, characterized by increased TNF-α–producing B cells, enhanced epithelial CD1d expression, B-cell and germinal-center hyperplasia, and disruption of the epithelial barrier associated with resident commensal bacteria (137). Tonsillar B cells were predominantly IgG/IgM-positive, with a smaller population of IgA-positive cells localized to the follicular margins. These findings support local tonsillar B-cell activation but do not establish systemic immunoglobulin dysfunction or determine whether these immune alterations are attributable specifically to OSA rather than to underlying adenotonsillar inflammation. Beyond local tonsillar immunity, IgM has also been investigated in the context of OSA-associated renal injury. In an adult OSA cohort, urinary IgM levels were associated with both the oxygen desaturation index and apnea–hypopnea index, and these associations remained significant after adjustment for several potential confounders (138). Because IgM is a large pentameric molecule, its presence in urine likely reflects impairment of the glomerular filtration barrier rather than systemic IgM dysregulation. Urinary IgM should therefore be interpreted primarily as a potential marker of OSA-associated glomerular injury rather than direct evidence of altered antibody production. Overall, current studies do not demonstrate a consistent OSA-specific immunoglobulin profile.

### 5.2 Complement system in OSA

The complement system is one of the most extensively studied components of innate immunity. It comprises a network of plasma proteins activated through sequential proteolytic cascades, promoting pathogen clearance and inflammatory signaling. Dysregulation of complement pathways has been implicated in autoimmune diseases, neurodegenerative disorders, sepsis and cancer. Despite its central role in immune regulation, relatively few studies have investigated complement activation in relation to either sleep disturbance or OSA.

#### 5.2.1 Evidence from sleep deprivation models

One small human study reported increased circulating complement components C3 and C4 not C3 and C5 following 24-hour sleep deprivation (128). Reis et al. (2011) demonstrated that C3a normally increases during nighttime sleep in healthy individuals, whereas plasma C3 and C4 decrease (139). Continuous wakefulness abolished the physiological nocturnal increase in C3a. A cross-sectional study of 36 healthy young men also linked polysomnographic sleep measures with complement regulation. Higher C3 and C4 were associated with a greater proportion of REM sleep, whereas lower C1-inhibitor and complement factor I were associated with reduced slow-wave sleep (140). In rats, 96 hours of paradoxical sleep deprivation increased circulating C3 concentrations (134). These studies examined acute sleep deprivation, physiological sleep, or experimental animal models rather than patients with OSA. Thus, although they suggest that sleep and circadian state can modulate complement levels, they do not establish that OSA produces similar alterations. In particular, the effects of continuous wakefulness should not be extrapolated directly to OSA, which is characterized by recurrent upper-airway obstruction, intermittent hypoxia (IH), and sleep fragmentation (SF).

#### 5.2.2 Direct evidence from patients with OSA

Wang et al. (2018) reported significantly higher serum C3 and total complement activity in patients with moderate-to-severe OSA than in participants with mild or no OSA (141). One month of CPAP treatment significantly reduced C3 levels (141), suggesting that complement activation may be at least partially reversible with OSA treatment. However, these findings derive from a limited adult clinical evidence base and the short treatment period, and observational comparisons do not establish that complement activation directly mediates OSA-related metabolic or vascular complications. Current evidence supports a possible relationship among OSA, IH, complement activation, endothelial dysfunction, oxidative stress and vascular injury. Nevertheless, proposed mechanisms involving C3a, CD59, endothelial permeability and TLR4/NF-κB signaling have not all been directly demonstrated in human OSA cohorts and should not be presented as established OSA-specific pathways without appropriate supporting evidence. The available direct evidence suggests that complement activation may occur in moderate-to-severe OSA, whereas the role of mucosal and systemic immunoglobulins remains poorly defined. Conclusions should remain cautious because humoral-immunity research in OSA is characterized by limited numbers of studies, small cohorts, substantial reliance on pediatric populations and limited adult data. Larger longitudinal adult studies and treatment response studies are needed to differentiate OSA specific immune alterations from the effects of experimental sleep loss, obesity, adenotonsillar disease, age and associated comorbidities.

### 5.3 Cell adhesion molecules and endothelial activation

Cell adhesion molecules play a critical role in leukocyte–endothelial interactions and the regulation of immune cell trafficking. Several studies have examined the effects of sleep deprivation on circulating soluble adhesion molecules, including E-selectin, vascular cell adhesion molecule-1 (VCAM-1) and intercellular adhesion molecule-1 (ICAM-1) in healthy individuals (142). Following one night of sleep deprivation, E-selectin and ICAM-1 levels increased, whereas VCAM-1 remained unchanged. These findings were interpreted as evidence of endothelial activation and enhanced pro-inflammatory activity, potentially linking sleep disruption to cardiovascular disease risk (142). Global leukocyte gene expression studies further support the pro-inflammatory effects of sleep disruption. Prolonged sleep deprivation has been shown to upregulate inflammatory signaling pathways (143) and impair leukocyte function (144), reinforcing the concept that chronic sleep fragmentation in OSA contributes to sustained immune dysregulation and systemic inflammation. **Figure 2** illustrates the alterations in innate, adaptive and humoral immune responses associated with OSA.

**Figure 2:**
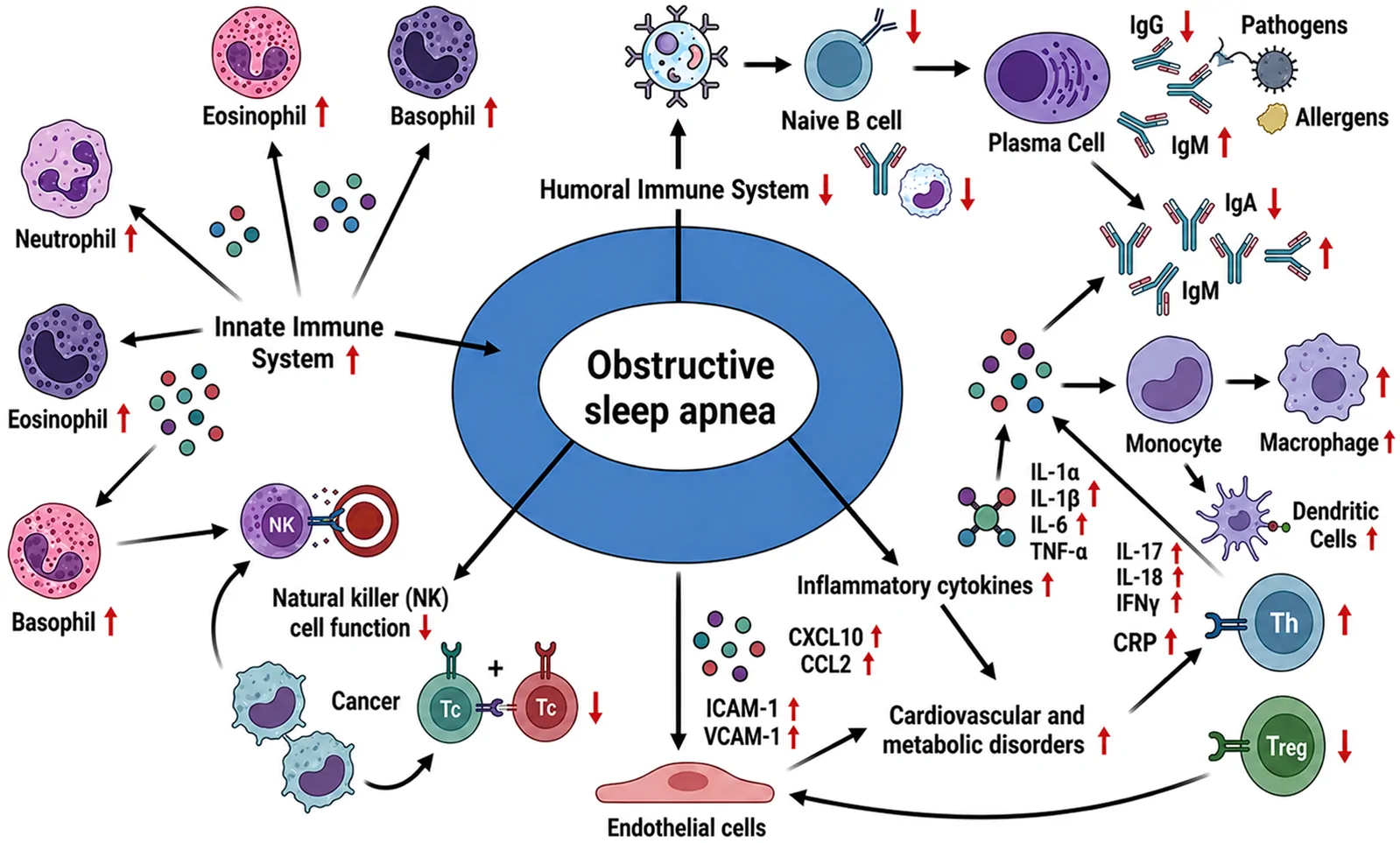
Immunological alterations are potentially associated with obstructive sleep apnea (OSA). OSA is associated with broad dysregulation of both innate and adaptive immune responses. Within the innate immune system, OSA is linked to increased numbers and activation of neutrophils, eosinophils, basophils, monocytes, macrophages and dendritic cells, accompanied by elevated production of pro-inflammatory cytokines, including IL-1α, IL-1β, IL-6, TNF-α, CXCL10, and CCL2. OSA also impairs natural killer (NK) cell function and alters T-cell homeostasis, characterized by increased T-helper (Th) cell activity, reduced regulatory T-cell (Treg) populations, and changes in cytotoxic T-cell responses. In the humoral immune system, OSA affects B-cell differentiation and antibody production, resulting in altered immunoglobulin profiles, including reduced IgG and IgA levels and increased IgM levels. These immune alterations contribute to systemic inflammation, endothelial dysfunction and the development of cardiovascular, and metabolic complications. Impaired immune surveillance may also influence cancer risk and progression. Red arrows indicate increases, whereas downward arrows indicate decreases in cell populations, immune functions, or circulating mediators associated with OSA.

## 6 OSA and inflammation

Inflammation disrupts the restoration of physiological homeostasis and contributes to the development of several chronic disorders, including type 2 diabetes, obesity, cardiovascular disease, asthma, chronic pain, cancer and neurodegenerative diseases. Aging is associated with a gradual increase in inflammatory biomarkers, a phenomenon termed *inflammaging* (145). Unlike classical acute inflammation, inflammaging is characterized by a persistent, low-grade elevation of inflammatory mediators in the absence of overt local inflammatory signs. This condition, commonly referred to as low-grade inflammation, is typically assessed using markers such as white blood cell (WBC) count, neutrophil count, C-reactive protein (CRP), interleukin-6 (IL-6) and platelet counts, often demonstrating a two- to three-fold increase above baseline levels (146). Low-grade inflammation contributes to endothelial dysfunction, atherosclerosis, insulin resistance and neuroinflammation (147–149), while also impairing adaptive immune responses through alterations in T-cell, B-cell, and dendritic cell functions (150, 151). Consequently, inflammation is increasingly recognized as both a consequence and driver of immune dysfunction. Collectively, available evidence suggests that OSA and associated sleep disturbances promote low-grade systemic inflammation, which may further impair immune function and contribute to a self-perpetuating cycle of inflammation and immune dysregulation (22, 29, 33–35, 152).

### 6.1 Low grade inflammation associated with OSA

Although direct evidence linking OSA to low-grade inflammation remains limited. The experimental studies have shown that sleep deprivation and sleep fragmentation can activate inflammatory pathways, enhance leukocyte recruitment and stimulate the production of pro-inflammatory mediators. Several mechanisms have been proposed to underlie these effects, including increased exposure to environmental stimuli, activation of damage-associated molecular patterns (DAMPs) and pathogen-associated molecular patterns (PAMPs), stimulation of pattern-recognition receptors (PRRs), oxidative stress–induced cellular injury, and dysregulation of the hypothalamic–pituitary–adrenal (HPA) axis. Collectively, these processes may contribute to sustained immune activation and chronic low-grade inflammation (153–156). Furthermore, several comorbid and environmental factors frequently associated with OSA, including obesity, circadian disruption, unhealthy lifestyle behaviors, psychosocial stress and socioeconomic determinants of health, may contribute to heightened inflammatory activity. These factors should be carefully considered when evaluating immune dysregulation and inflammatory changes attributed to OSA (157).

Clinical and experimental studies further support the presence of systemic inflammation in OSA. A meta-analysis of 102 studies demonstrated significantly elevated circulating inflammatory mediators in both adult and pediatric OSA populations, supporting a role for inflammation in disease pathophysiology and progression (21). In addition, elevated pan-immune-inflammation values (PIV) have been independently associated with increasing disease severity as measured by the apnea–hypopnea index (AHI) (158). Other studies have reported increased CRP/hs-CRP, lipopolysaccharide (LPS) and markers of intestinal barrier dysfunction, suggesting contributions from microbial translocation and gut-derived inflammation (159). Furthermore, Mendelian randomization analyses have implicated several inflammatory proteins, cytokine receptors and chemokines in OSA susceptibility and progression, providing additional evidence for a mechanistic role of inflammation in OSA pathogenesis (22). Experimental studies have demonstrated a bidirectional relationship between sleep and immune signaling. While numerous cytokines participate in the physiological regulation of sleep, sleep deprivation, sleep fragmentation, and IH can, in turn, disrupt immune homeostasis and modulate inflammatory pathways (160–163). The roles of specific inflammatory mediators and cytokines implicated in OSA are discussed in the following section.

## 7 Cytokines and cytokine production in OSA

### 7.1 TNF-α

Tumor necrosis factor-alpha (TNF-α) is a key pro-inflammatory cytokine that mediates immune and inflammatory responses in OSA, particularly in response to IH and SF (164, 165). TNF-α contributes to systemic inflammation, daytime sleepiness, endothelial dysfunction and cardiometabolic complications. It has been implicated in the pathogenesis of OSA in both adults and children (62, 112, 166). OSA severity has been associated with elevated circulating inflammatory cytokines, particularly TNF-α and IL-6 (167).

The regulatory role of TNF signaling has also been investigated through its soluble receptors. Levels of soluble TNF receptor I (sTNFRI), a modulator and inhibitor of TNF signaling, increased after four nights of total sleep deprivation, potentially reflecting a compensatory response to elevated TNF-α production by activated leukocytes (168). Experimental studies investigating the impact of sleep loss on TNF-α have reported variable results. In healthy adults, plasma TNF-α concentrations were largely unaffected during or immediately after acute sleep deprivation (129, 169, 170). However, lower TNF-α levels were observed during sleep and higher levels during nocturnal wakefulness, suggesting that TNF-α is subject to homeostatic regulation (171). Similarly, modest sleep restriction to 6 hours per night for one week increased circulating TNF-α levels (172), indicating that prolonged sleep loss may promote inflammatory activation. Consistent with this concept, several studies have reported increased TNF-α levels following chronic sleep restriction and OSA-related sleep disturbances (159, 173, 174). In contrast, some investigations have observed reduced TNF-α levels in OSA patients, highlighting variability among study populations and methodologies (118, 175). A recent study involving children with obstructive sleep apnea-hypopnea syndrome (OSAHS) reported significantly reduced circulating TNF-α levels compared with controls, with levels improving following surgical treatment (118). In contradiciton with such findings, higher circulating morning TNF-α levels in children with OSA which were associated with excessive daytime slepeiness scores and reductions in such levels after treatment have also been reported (176). Of note, lower TNF-α levels were observed during sleep and higher levels during nocturnal wakefulness, suggesting that TNF-α is subject to homeostatic regulation (101).

Animal studies have likewise demonstrated tissue-specific alterations in TNF-α expression following sleep disruption and IH. Sleep fragmentation increased TNF-α expression in cardiac tissue as early as one hour after exposure, whereas splenic TNF-α expression remained unchanged (177). Similarly, disrupted sleep even in the absence of insufficient sleep leads to higher levels in mice (178, 179). Chronic intermittent hypoxia significantly increased pulmonary TNF-α expression (180), while elevated serum TNF-α levels were observed in rats with heart failure exposed to intermittent hypoxia, particularly in the SF+IH group (181). In addition, sleep fragmentation increased joint TNF-α expression and delayed resolution of inflammation in a Lyme arthritis mouse model (182). Collectively, individual studies report inconsistent TNF-α findings in OSA, ranging from reduced or unchanged levels to significant elevations. This variability may reflect differences in OSA severity, age, sampling time, comorbidities, treatment modality, and assay methodology. Nevertheless, meta-analytic evidence from randomized trials indicates that CPAP modestly but significantly reduces TNF-α levels, supporting an overall association between OSA and TNF-α–related inflammation despite heterogeneity across individual studies (175).

### 7.2 IL-1

Interleukin-1 (IL-1), particularly IL-1β, is a key pro-inflammatory cytokine that has been extensively studied in the context of sleep deprivation and obstructive sleep apnea (OSA). Experimental and clinical evidence suggests that IL-1 plays an important role in both sleep regulation (23, 183–185) and inflammatory responses; however, findings have been somewhat inconsistent. In humans, acute sleep disruption appears to induce transient increases in IL-1 levels, as demonstrated following a single night of total sleep deprivation (159, 164, 186).

Prolonged sleep deprivation protocols, including two consecutive nights of total sleep deprivation and selective REM sleep deprivation, did not result in significant changes in circulating IL-1 levels in healthy adults (129). Consistent with these variable findings, animal studies have also yielded mixed results, with some demonstrating increased plasma IL-1 levels following sleep deprivation (187), while others reported no significant alterations (188).

At the cellular level, neither IL-1 production nor IL-1 mRNA expression in peripheral blood leukocytes appears to be consistently altered by acute sleep deprivation in humans. Nevertheless, prolonged sleep deprivation has been associated with increased IL-1 mRNA expression in both humans and animal models (115, 187), suggesting that transcriptional changes may precede detectable systemic alterations. Importantly, although studies differ regarding the magnitude of IL-1 responses, none have reported reduced IL-1 levels following sleep deprivation or sleep restriction indicating a tendency toward maintenance or activation of pro-inflammatory signaling. The endogenous inhibitor of IL-1, the IL-1 receptor antagonist (IL-1ra), has been reported to increase following sleep deprivation in both humans and rodents (187). This increase may represent a homeostatic compensatory mechanism aimed at limiting excessive IL-1 activity and restoring inflammatory balance.

Evidence from OSA studies supports a role for IL-1β in disease-associated inflammation. Elevated IL-1β levels have been reported in OSA patients, with concentrations increasing with disease severity, positively correlating with the apnea-hypopnea index (AHI), and negatively correlating with mean oxygen saturation, suggesting a contribution of IH to IL-1β-mediated inflammatory responses However, not all studies have observed significant differences in circulating IL-1α or IL-1β levels between OSA patients and controls (173). dditionally, increased CD16^+^ monocyte populations, particularly in OSA patients with higher body mass index (BMI), have been associated with enhanced IL-1β production, further supporting a role for innate immune activation in OSA-associated inflammation (101).

Animal studies provide further evidence that sleep disruption and IH induce tissue-specific IL-1β responses. Sleep fragmentation (SF) has been shown to increase IL-1β expression in multiple tissues, including adipose tissue, the heart, hypothalamus and other brain regions, although the magnitude of these effects varies by tissue (177). Similarly, IH exposure increases IL-1β expression in the hippocampus, brain and lung tissues, indicating an early neuroinflammatory and inflammasome-mediated response (189). Moreover, chronic IH elevates circulating IL-1β levels in mice, supporting the presence of systemic inflammation associated with IH (190). Overall, current evidence supports an important role for IL-1, particularly IL-1β, in inflammatory responses associated with sleep disturbance and OSA.

Although findings for circulating IL-1β levels are inconsistent, most clinical and experimental studies report increased IL-1β expression, enhanced transcriptional activity, or activation of IL-1–related signaling in response to sleep fragmentation and intermittent hypoxia. Consistent with these findings, meta-analyses by Yi et al. and Janmohammadi et al. reported significantly higher circulating IL-1β levels in patients with OSA than in controls (63, 191). While, Yi et al. and Mohammadi et al. showed a reduction in IL-1β levels following CPAP treatment (63, 192). Discrepancies among individual studies may reflect differences in sleep-loss duration, OSA severity, sampling time, participant characteristics, and assay methodology. Importantly, the Mendelian-randomization analysis by Yi et al. did not support a causal relationship between OSA and IL-1β. Collectively, current evidence supports an association between IL-1 signaling and OSA-related immune dysregulation and systemic inflammation, but a causal role has not been established.

### 7.3 IL-2

Interleukin-2 (IL-2) is an immunoregulatory cytokine primarily secreted by activated CD4^+^ T cells and is essential for T-cell proliferation differentiation and immune tolerance (193). IL-2 also participates in neuroimmune interactions and has been implicated in sleep regulation through its promotion of non-rapid eye movement (NREM) sleep and modulation of interactions between immune activation and sleep (194). Given its central role in adaptive immunity, IL-2 has been investigated in the context of sleep deprivation and obstructive sleep apnea (OSA), both of which are associated with immune dysregulation and IH. However, compared with other inflammatory cytokines, evidence regarding the role of IL-2 in OSA remains limited and inconsistent (118). Recent evidence suggests that OSA particularly in children may be associated with reduced IL-2 levels (118). In a study of children with OSA, circulating IL-2 and IFN-γ levels were significantly lower than those of healthy controls (118). Importantly, surgical treatment resulted in significant improvements in both IL-2 levels and immune-cell profiles at 3- and 6-month follow-up, suggesting that IL-2 suppression may be reversible and related to disease severity. Collectively, the available evidence suggests that IL-2 may be downregulated in OSA, potentially reflecting impaired T-cell function and adaptive immune dysregulation. However, the current evidence base is limited and is derived largely from pediatric populations. Additional studies are therefore required to determine whether similar alterations occur in adults and across different levels of OSA severity.

### 7.4 IL-4

Interleukin-4 (IL-4) is an anti-inflammatory cytokine primarily associated with Th2 immune responses and plays an important role in humoral immunity and maintenance of inflammatory balance (195). IL-4 also participates in sleep regulation by suppressing pro-inflammatory mediators modulating neuroimmune interactions and influencing normal sleep architecture. Experimental studies have shown that IL-4 inhibits spontaneous sleep *in vivo* models (161). Given its role in regulating Th1/Th2 immune balance, IL-4 has been investigated in OSA, a condition characterized by IH and chronic inflammation that may alter adaptive immune responses (122). Current evidence suggests that IL-4 is reduced or dysregulated in OSA, potentially contributing to a shift toward a pro-inflammatory immune profile (118). For example, a recent study of children with OSA reported significantly lower IL-4 levels compared with healthy controls, indicating impaired cytokine regulation and immune dysfunction (118). Similarly, studies evaluating cytokine ratios such as IFN-γ/IL-4 have demonstrated a shift toward pro-inflammatory responses, further supporting an imbalance between Th1 and Th2 immune activity in OSA (60). Reduced IL-4 levels may limit its anti-inflammatory and immunoregulatory functions thereby permitting sustained activation of pro-inflammatory pathways (112, 196). Experimental studies have produced somewhat contrasting findings. Intermittent hypoxia increased IL-4-producing CD4^+^ T cells in the spleen, suggesting altered T-helper cell polarization (126). In contrast, sleep fragmentation significantly reduced joint IL-4 expression in a murine Lyme arthritis model, indicating suppression of anti-inflammatory and tissue-repair mechanisms that normally contribute to the resolution of inflammation (182).

The available evidence suggests that IL-4 may contribute to immune dysregulation in OSA through alterations in the Th1/Th2 balance (195), however it does not establish a consistent direction of change or support IL-4 as a reliable biomarker of treatment response. The current body of evidence remains limited and, in some cases, contradictory. Consistent with this uncertainty, the meta-analysis by Mohammadi et al. found no significant change in circulating IL-4 following CPAP treatment (192). Differences in patient populations, OSA severity, experimental models, and study design may contribute to these inconsistencies. Further studies are needed to clarify the role of IL-4 in OSA pathophysiology and determine whether it has utility as a biomarker of immune dysregulation or disease progression.

### 7.5 IL-6

Interleukin-6 (IL-6) is a pleiotropic pro-inflammatory cytokine. It plays critical role in immune regulation, inflammation and neuroimmune signaling. Its involvement in sleep deprivation and obstructive sleep apnea (OSA) has been extensively investigated; however, findings regarding circulating IL-6 responses to sleep loss have been variable and sometimes inconsisten (146). Several studies have reported increased circulating IL-6 levels following sleep deprivation and in patients with OSA (58, 62, 63, 159, 186, 197, 198), whereas others have reported decreased levels (175, 199, 200) or no significant changes (201, 202). Despite these inconsistencies, most evidence suggests that prolonged sleep loss is associated with increased IL-6 levels, supporting its role as a marker of systemic inflammation (115, 122, 173, 203). Clinical studies consistently support an association between IL-6 and OSA-associated inflammation. Plasma IL-6 levels have been reported to be elevated in OSA patients compared with healthy controls (58, 173). Increased IL-6 levels have been reported, suggesting a pro-inflammatory shift in immune responses accompanied by immune cell dysfunction, including natural killer (NK) cell dysregulation (60). More recently, IL-6 protein and mRNA expression levels in peripheral blood mononuclear cells (PBMCs) were found to be significantly elevated in patients with OSA, with the highest levels observed in individuals with severe disease (29). Importantly, IL-6 levels decreased following positive airway pressure (PAP) therapy indicating that OSA-associated inflammatory alterations may be partially reversible (29). Beyond systemic inflammation, IL-6 may also contribute to OSA-associated neurocognitive dysfunction. Elevated serum IL-6 concentrations have been associated with poorer cognitive performance increased daytime sleepiness and reduced oxygen saturation during sleep (204). OSA patients with cognitive impairment exhibited higher IL-6 levels than those without cognitive deficits suggesting that IH may promote neuroinflammation through IL-6-dependent mechanisms (204). These findings support the potential utility of IL-6 as a biomarker linking inflammation hypoxia and neurocognitive impairment in OSA.

Extensive sleep deprivation has been shown to increase intracellular IL-6 production and IL-6 mRNA expression in peripheral blood lymphocytes in both humans and animals (205). Similarly, elevated IL-6 protein and mRNA expression has been observed in multiple tissues, including the brain, in several *in vivo* studies, supporting a potential role for IL-6 in the development of neuroinflammatio (206, 207). These findings indicate that transcriptional and intracellular changes may represent more sensitive indicators of sleep-induced immune activation than circulating protein concentrations alone. Animal studies further highlight the contribution of IL-6 to sleep disruption and IH-induced inflammation. Recent evidence demonstrates that acute sleep fragmentation (SF) induces a rapid increase in serum IL-6 levels in male mice, with significant elevations detected as early as 6 hours and persisting through 24 hours after exposure (177). Elevated plasma IL-6 levels have also been observed following 20 weeks of chronic sleep fragmentation (SF), supporting a role for sustained sleep disruption in promoting persistent systemic inflammation in animal models (110). Enhanced IL-6 responses to lipopolysaccharide challenge have been reported in IH models, indicating that IH may alter innate immune responsiveness and promote a heightened inflammatory state (189). Pulmonary IL-6 expression was also increased following IH exposure in mice (208). Consistent with these findings, *in vitro* IH significantly increased IL-6 mRNA expression and IL-6 secretion indicating activation of inflammatory signaling pathways (209). Elevated serum IL-6 concentrations have likewise been reported following chronic intermittent hypoxia (CIH) exposure *in vivo* supporting a role in neuroimmune dysfunction (190). Furthermore, chronic IH increased IL-6 expression in CD11b-DTR mice whereas depletion of CD11b^+^ myeloid cells attenuated this response implicating myeloid cells as an important source of IH-induced IL-6 production (103). In a heart failure plus IH model, elevated serum IL-6 levels were associated with delayed polymorphonuclear neutrophil apoptosis and worsened cardiac dysfunction further supporting its role in hypoxia-induced inflammatory injury (181).

Although individual studies have shown decreased, increased or unchanged circulating IL-6 levels, while meta-analyses findings seem more consistent. Yi et al. found that IL-6 was elevated in OSA compared with controls and decreased following effective treatment (63). The meta-analyses by Zhu et al. and Mohammadi et al. demonstrated significant reductions in IL-6 after CPAP therapy (175, 192). Discrepancies among individual studies may reflect differences in OSA severity, duration and type of sleep disruption, sampling time, participant characteristics and assay methodology. However, the Mendelian-randomization analysis by Yi et al. did not establish a causal relationship between OSA and IL-6. Thus, the evidence supports IL-6 as a marker of OSA-associated systemic inflammation and treatment response, although its causal role remains uncertain.

### 7.6 IL-8

Interleukin-8 (IL-8) is a pro-inflammatory chemokine that serves as a potent mediator of inflammation through the recruitment and activation of neutrophils. In addition, IL-8 contributes significantly to vascular inflammation. It is predominantly secreted by monocytes/macrophages, epithelial cells and endothelial cells in response to inflammatory stimuli (210). Biologically, IL-8 functions as a potent chemoattractant and activator of neutrophils, stimulating degranulation and the release of proteolytic enzymes and reactive oxygen species. These processes can promote tissue injury, oxidative stress and endothelial dysfunction, thereby contributing to the progression of inflammatory disease (211, 212). In OSA, IL-8 is widely recognized as a marker of low-grade systemic inflammation and IH. Recurrent cycles of hypoxia and reoxygenation are believed to induce inflammatory pathways that enhance IL-8 release, contributing to vascular injury and cardiovascular dysfunction associated with the disease (30, 39).

Accumulating clinical evidence indicates that circulating IL-8 levels are elevated in patients with OSA, supporting a role for IL-8 in the systemic inflammatory response associated with the disorder (62, 63). A systematic review of 16 studies consistently demonstrated significantly higher serum IL-8 concentrations in OSA patients compared with healthy controls (65, 66, 165, 213–225). These findings are further supported by meta-analyses that reported significantly elevated IL-8 concentrations in OSA patients (39, 226). However, despite the consistency of these findings, whether IL-8 can serve as a diagnostic or predictive biomarker for OSA remains uncertain. Previous meta-analyses were constrained by small sample sizes, a limited number of studies with a lack of subgroup and meta-regression analyses, limiting confidence in the clinical utility of IL-8. Additionally, differences in study design and patient populations may contribute to the heterogeneity of findings. The role of IL-8 has also been explored in pediatric OSA. Elevated IL-8 levels have been reported in children with OSA, particularly in morning blood samples. Notably, higher IL-8 concentrations were associated with reduced pulse transit time (PTT), suggesting increased vascular stiffness and the presence of early vascular alterations. However, compensatory physiological mechanisms in children may partially mitigate or mask the clinical manifestations of these vascular changes (30).

Although evidence from animal models of OSA remains limited, *in vitro* studies support a mechanistic role for IL-8 in OSA-associated inflammation. Conditioned media derived from airway epithelial cells exposed to IH exhibited increased IL-8 production and enhanced neutrophil chemotaxis, indicating that IH may promote neutrophil-mediated inflammatory responses through IL-8 signaling (227). Although considerable evidence supports a role for IL-8 in OSA-associated inflammation and vascular dysfunction, methodological heterogeneity and the lack of longitudinal studies limit its utility as a standalone biomarker. Future studies using standardized protocols and integrated cytokine profiling are needed to clarify the contribution of IL-8 to OSA pathophysiology and its potential clinical relevance.

### 7.7 IL-10

Interleukin-10 (IL-10) is a key anti-inflammatory cytokine that plays a critical role in regulating and limiting immune responses, thereby maintaining immune homeostasis. It exerts its effects by inhibiting the production of pro-inflammatory cytokines and suppressing T-cell proliferation. IL-10 is particularly important in the context of sleep disturbances and obstructive sleep apnea (OSA), where it serves as a regulatory cytokine that counterbalances the inflammatory responses induced by IH (62, 228). Clinical evidence suggests that IL-10 levels are generally reduced in OSA contributing to a shift toward a pro-inflammatory immune profile. Reduced plasma IL-10 levels have been observed in patients with obstructive sleep apnea (OSA), reflecting impaired anti-inflammatory regulation. This decrease is associated with a shift toward a pro-inflammatory immune profile and may contribute to the systemic inflammation and increased cardiovascular risk characteristic of OSA (173, 229). Additional evidence for IL-10 dysregulation has emerged from studies examining immune balance and T-cell subsets. Reduced frequencies of regulatory T (Treg) cells and lower IL-10 levels, accompanied by enhanced Th17-cell responses, have been observed in children with OSA, resulting in a markedly increased Th17/Treg ratio. These immune alterations are more pronounced in children with coexisting allergic rhinitis, suggesting that additional inflammatory conditions may further exacerbate immune dysregulation in OSA (174). Furthermore, a reciprocal relationship between IL-6 and IL-10 emerged in non-obese children with OSA which was abrogated after treatment with IL-10 rising and IL-6 declining after adenotonsillectomy (230). Experimental studies in animals further support a role for IL-10 in OSA-associated immune regulation. Chronic IH enhanced lipopolysaccharide-induced IL-10 expression suggesting modification of immune regulatory pathways following prolonged hypoxic exposure (189). In animal models, IH altered IL-10 expression and CD11b^+^ cell depletion significantly modulated IL-10 levels indicating a role for macrophage populations in regulating anti-inflammatory responses during IH exposure (103). In addition, chronic sleep restriction in mice significantly reduced IL-10 expression in B-1 cells suggesting impaired anti-inflammatory and immunoregulatory function (231).

Overall, individual clinical studies generally demonstrate reduced levels of IL-10 in OSA, although findings from treatment-related and experimental studies show variability. Consistent with the clinical findings, the meta-analyses by Yi et al. and Janmohammadi et al. found significantly lower circulating IL-10 levels in patients with OSA than in controls (63, 191). However, Mohammadi et al. found no significant change in IL-10 following CPAP therapy, contrasting with the increased IL-10 reported after adenotonsillectomy in an individual pediatric study (192). These discrepancies may mirror differences in OSA severity, age, comorbidities, treatment modality and duration, and the cellular and circulating or tissue-specific source of IL-10. A reduction in IL-10 may be frequently associated with diminished regulatory T-cell activity, may impair immune homeostasis and promote persistent inflammation in OSA. However, its role in disease pathogenesis and its reliability as a therapeutic, prognostic or treatment-response biomarker require further investigation in animal and longitudinal clinical studies.

### 7.8 IL-12

Interleukin-12 (IL-12) is a pro-inflammatory cytokine produced primarily by antigen-presenting cells that plays a central role in promoting Th1 immune responses and stimulating interferon-γ production. In addition to its immunoregulatory functions, IL-12 contributes to the regulation of sleep–wake cycles by modulating non-rapid eye movement (NREM) sleep and interacting with circadian immune mechanisms, highlighting the bidirectional relationship between sleep and immune function (228). Given its role in inflammatory and adaptive immune responses, IL-12 has been investigated as a component of the cytokine network activated by IH and systemic inflammation in OSA. Current evidence suggests that IL-12 levels are elevated in OSA, with a recent meta-analysis demonstrating significantly higher circulating IL-12 concentrations in patients compared with healthy controls, supporting the presence of a systemic pro-inflammatory state associated with the disorder (63). Multiple studies have reported elevated IL-12 levels in patients with OSA compared with healthy controls. However, Mendelian randomization analyses have not supported a causal relationship between OSA and IL-12 levels, suggesting that increased IL-12 may reflect the systemic inflammatory state associated with the disorder rather than a direct pathogenic mechanism (63). Current evidence indicates that IL-12 levels are elevated in OSA, suggesting activation of pro-inflammatory immune pathways. However, the available data are limited and rely largely on meta-analyses rather than mechanistic or longitudinal studies. The lack of causal evidence and functional investigations restricts the clinical utility of IL-12 as a biomarker. Further well-designed clinical and experimental studies are needed to clarify its role in OSA-associated immune dysregulation and disease pathogenesis (63).

### 7.9 IL-17

Interleukin-17 (IL-17) is a pro-inflammatory cytokine primarily produced by T helper 17 (Th17) cells and plays a critical role in neutrophil recruitment and the amplification of inflammatory responses. In OSA, IL-17 has emerged as a key mediator of immune dysregulation, contributing to the chronic systemic inflammation induced by IH. Elevated IL-17 levels have been associated with increased inflammatory activity and may play an important role in the pathogenesis of OSA-related comorbidities (118). Clinical studies consistently demonstrated elevated IL-17 levels in OSA patients. In a cohort of 46 male subjects categorized into control, mild, moderate and severe OSA groups, serum IL-17A levels increased progressively with disease severity (232). Similarly, a study comparing 92 patients with severe OSA and 30 age-, sex- and BMI-matched controls reported significantly elevated serum IL-17 levels in the OSA group (233). In a related study, IL-17 levels were negatively correlated with vitamin D concentrations in patients with severe OSAHS, highlighting a potential link between IH, immune dysregulation and IL-17 activity (234). Similarly, patients with severe OSA exhibited significantly elevated IL-17 levels compared with controls (235).

Elevated IL-17 levels have also been observed in pediatric OSA. A study involving 47 non-obese children with OSA and 32 healthy controls found significantly higher serum IL-17 levels in affected children, with increased IL-17 concentrations correlating with poorer neurocognitive performance, suggesting a potential contribution to OSA-related cognitive dysfunction (236). Importantly, obesity was excluded and BMI was included among the covariates in regression analyses, with IL-17 remaining significantly associated with OSA severity, supporting an OSA-related inflammatory effect independent of obesity. Similarly, among obese children undergoing polysomnographic evaluation, IL-17 was identified as a key inflammatory cytokine associated with both mild and moderate-to-severe OSA (237), suggesting that OSA-related IL-17 dysregulation is also evident within an obese population. Meta-analyses have consistently demonstrated significantly elevated IL-17 levels in both adult and pediatric populations with OSA (191). Golshah et al. (2024) also observed a similar trend toward increased IL-17 levels through meta-analysis (238). Additional evidence for IL-17 dysregulation has emerged from studies examining Th17/Treg balance. In a cohort of 56 patients with OSA and 40 healthy controls, Th17-cell frequencies and serum IL-17 levels were significantly elevated in individuals with OSA (29).

Animal studies further support the involvement of IL-17 in OSA-related inflammation. Exposure of male C57BL/6N mice to IH for four weeks resulted in increased IL-17A-producing CD4^+^ T cells, consistent with enhanced Th17-driven inflammatory responses (126). Following a four-week normoxic recovery period these alterations returned to control levels suggesting that IH directly drives IL-17-associated immune changes (126). Similarly, another study used a rat model of experimentally induced OSA and showed increased IL-17-producing cells in the brain cecum and ileum (239).

The current evidence establishes IL-17 as a vital mediator of immune dysregulation and inflammation in OSA. Consistent with individual clinical studies, the meta-analyses conducted by Janmohammadi et al. and Golshah et al. reported significantly increased IL-17 levels in adults and children with OSA (191, 238). However, treatment-related results seem less consistent. The meta-analysis by Mohammadi et al. reported no significant reduction in circulating IL-17 following CPAP (192). This discrepancy is supposed to be due to differences in disease severity, treatment duration and CPAP adherence. Thus, increased IL-17 is associated with OSA severity, hypoxemia and inflammatory sequelae, while experimental studies support its involvement in cardiovascular, neuroinflammatory and immune dysfunction. Further longitudinal studies are required to clarify their pathogenic role.

### 7.10 IL-18

Interleukin-18 (IL-18), a member of the IL-1 cytokine family, is a potent pro-inflammatory mediator involved in Th1-associated immune responses. Accumulating evidence indicates that IL-18 levels are elevated in OSA and may contribute to the systemic inflammatory burden driven by IH and disease progression (240). Clinical evidence consistently indicates increased IL-18 levels in OSA. A study of 52 male patients with OSA and 18 healthy controls demonstrated a progressive rise in plasma IL-18 concentrations with increasing disease severity. Furthermore, IL-18 levels decreased significantly after 90 days of CPAP treatment, suggesting a link between IL-18 expression and OSA-related pathophysiology (240). Likewise, a study involving 66 patients with OSA demonstrated significantly higher IL-18 levels than those observed in healthy controls, with IL-18 concentrations increasing in parallel with disease severity (241). In addition, patients with preeclampsia and coexisting OSA exhibited significantly elevated serum IL-18 levels compared with controls, with concentrations decreasing significantly following CPAP therapy (242). Further evidence indicates that IL-18 levels are increased in patients with OSA and may contribute to the development of OSA-associated renal dysfunction, highlighting a potential role for IL-18 in end-organ complications of the disease (243). Experimental evidence also implies IL-18 in OSA-related inflammatory responses. CIH induced significant upregulation of IL-18 in neural and pulmonary tissues of wild-type mice, whereas these effects were substantially reduced in NLRP3 knockout animals, supporting a role for IL-18 as a downstream mediator of NLRP3 inflammasome activation (244). Previous studies have shown that sleep deprivation increases IL-18 expression in brain regions involved in sleep regulation, whereas IL-18 knockout mice display reduced sleep responses. Together, these observations suggest that IL-18 contributes to both sleep regulation and the inflammatory consequences of sleep loss (245, 246). Moreover, suppression of NLRP3 signaling has been shown to reduce IH–induced neuroinflammation, neuronal damage and respiratory dysfunction in animal models, further supporting the involvement of IL-18–related pathways in OSA-associated complications (247, 248). Overall, current evidence supports IL-18 as an important inflammatory mediator in OSA.

The available literature consistently reports elevated IL-18 levels in OSA, with reductions following CPAP treatment. These findings are supported by meta-analyses by Yi et al. and Golshah et al., which demonstrated higher IL-18 levels in patients with OSA, particularly in adults. Similarly, Mohammadi et al. confirmed elevated IL-18 levels in OSA and a significant reduction following CPAP treatment (63, 192, 238). However, the Mendelian-randomization analysis by Yi et al. did not establish a causal relationship between IL-18 and OSA (63). Differences across age groups and individual studies may reflect variations in OSA severity, comorbidities, treatment duration, and other clinical and methodological factors. Overall, IL-18 appears to be a potential marker of OSA-associated inflammation and treatment response. However, further mechanistic and longitudinal studies are needed to determine whether IL-18 contributes directly to OSA pathogenesis or primarily reflects underlying inflammatory activity.

### 7.11 ILs 35 and 37

Interleukin-35 (IL-35) and interleukin-37 (IL-37) are anti-inflammatory cytokines that play vital roles in immune regulation and maintenance of immune homeostasis (249). For example, IL-35 primarily regulates regulatory T-cell activity and suppresses excessive immune responses, whereas IL-37 exerts broad anti-inflammatory effects on both innate and adaptive immunity (249). In OSA, evidence regarding IL-35 and IL-37 remains sparse and their precise roles in disease pathophysiology have not been well characterized in either human or animal studies. In adults with OSA, serum IL-35 and IL-37 levels were significantly elevated and correlated with measures of disease severity. These findings suggest that increased expression of these anti-inflammatory cytokines may reflect a compensatory response to OSA-associated inflammation induced by IH and sleep disruption (250). IL-37 transgenic mice exhibited attenuated inflammation-induced NREM sleep responses following LPS and IL-1β administration and improved outcomes during influenza infection, suggesting that IL-37 modulates inflammatory sleep responses and limits immunopathology. However, no IH or SF models relevant to OSA were examined (251). Thus, IL-35 and IL-37 may participate in compensatory anti-inflammatory pathways in OSA. Further clinical and mechanistic studies are needed to clarify their roles and potential utility as biomarkers of OSA-associated immune dysregulation.

## 8 Other cytokines and signaling pathways

Although physiologically important, several other cytokines and immune pathways have been investigated albeit less extensively in OSA. These include PD-1/PD-L1 immune checkpoint activation, Toll-like receptor (TLR)-mediated inflammatory responses and NLRP3 inflammasome signaling. Collectively, these pathways contribute to systemic inflammation, immune dysregulation and the development of cardiovascular, and other OSA-related complications.

### 8.1 PD-1/PD-L1 immune checkpoints

Programmed cell death protein-1 (PD-1) and programmed death ligand-1 (PD-L1) are important regulators of T-cell activation and immune tolerance. Studies in patients with OSA have demonstrated increased PD-1 expression on CD4^+^ and CD8^+^ T cells and elevated PD-L1 expression on monocytes, findings that are associated with chronic immune activation and inflammatory dysregulation. These alterations suggest a potential role for PD-1/PD-L1 signaling in the immune dysfunction observed in OSA (101). However, current human evidence is derived predominantly from circulating peripheral immune cells and soluble blood markers, whereas data demonstrating PD-1/PD-L1 alterations in tissue resident immune or structural cells in patients with OSA remain scarce.

The increased number of monocyte–T cell aggregates may reflect enhanced cell-to-cell interactions, potentially altering PD-1/PD-L1-mediated immune regulation and contributing to chronic inflammatory processes (101). PD-L1 expression has been reported to correlate positively with BMI, AHI, the severity of hypoxemia and monocyte redistribution. Mechanistically, IH-induced hypoxia-inducible factor-1α (HIF-1α) may upregulate PD-L1 transcription through binding to hypoxia-response elements within the PD-L1 promoter (64, 166, 252). Increased monocyte–T cell interactions may further enhance PD-1/PD-L1-mediated immune dysregulation, thereby contributing to persistent inflammation in OSA. Notably, therapeutic interventions such as hypoglossal nerve stimulation have been associated with reduced PD-L1 expression, suggesting a partial restoration of immune homeostasis (253). Experimental studies have demonstrated that IH increases PD-L1 expression on F4/80^+^ cells and PD-1 expression on both CD4^+^ and CD8^+^ T cells. Similar findings were observed in cultured human peripheral blood mononuclear cells exposed to IH and oxidative stress, whereas sleep fragmentation alone did not produce significant changes. These results suggest that IH may be an important contributor to PD-1/PD-L1 pathway activation in OSA (254). However, the independent effects of SF remain insufficiently characterized, and the absence of a significant effect in a limited experimental model does not exclude immune-checkpoint modulation by chronic SF in patients with OSA. Further studies are needed to distinguish the respective contributions of IH and SF and determine whether these circulating immune alterations are also present in OSA-relevant tissues.

### 8.2 NF-κB

​ Nuclear factor-kappa B (NF-κB) is a key inflammatory transcription factor. It regulates the expression of numerous genes involved in immune activation, cytokine production and inflammatory responses. Increasing evidence indicates that NF-κB signaling is activated in OSA, primarily as a consequence of IH. It may contribute to both systemic and tissue-specific inflammation (64). Clinical evidence for NF-κB activation in OSA has been demonstrated in studies involving patients with varying degrees of disease severity, supporting a relationship between OSA and enhanced inflammatory signaling (165). OSA patients exhibit significantly elevated levels of TNF-α and IL-8, both of which correlate with the degree of nocturnal oxygen desaturation and decrease following continuous positive airway pressure (CPAP) therapy. These findings support the activation of NF-κB-regulated inflammatory pathways and highlight the contribution of IH to systemic inflammation in OSA (165). Experimental studies further support the activation of the NF-κB pathway during IH. Increased expression of NF-κB signaling components, including p65 and phosphorylated p65 (phospho-p65), has been observed following IH exposure, indicating enhanced activation of NF-κB-mediated inflammatory signaling (126). Similarly, increased NF-κB p65 expression has been observed in lung tissue following IH hypoxia exposure, further supporting a role for NF-κB activation in OSA-related inflammatory responses (208). Chronic IH also increased phosphorylated NF-κB p65 expression in the spinal dorsal horn indicating activation of inflammatory transcriptional pathways within neural tissues (190). Collectively, these findings indicate that IH activates NF-κB signaling, which may serve as a key molecular mechanism linking OSA to systemic inflammation and the development of cardiovascular, pulmonary, and neurocognitive complications.

### 8.3 Toll-like receptor signaling

Toll-like receptors (TLRs) are central mediators of innate immune responses that detect microbial and danger-associated signals and initiate inflammatory cascades. Emerging evidence indicates that aberrant TLR signaling, especially activation of the TLR4/NF-κB pathway, contributes to the persistent inflammatory state associated with OSA (38, 255–258). Clinical evidence indicates that TLR2 and TLR6 expressions are upregulated in immune cells from patients with obstructive sleep apnea (OSA). Their expression levels correlate with disease severity. Notably, these changes have been linked to epigenetic alterations, including differential DNA methylation within the TLR2 promoter and TLR6 gene body, highlighting a potential role for epigenetic mechanisms in mediating OSA-associated immune dysfunction and chronic inflammation (259, 260). Experimental studies provide further evidence for the involvement of TLR signaling in OSA-related inflammation. IH increases pulmonary TLR4 expression, while TLR4 deficiency significantly reduces the associated inflammatory response. These observations suggest that activation of the TLR4 pathway is a critical driver of IH-induced pulmonary inflammation (208). In contrast, chronic sleep restriction has been shown to decrease the expression of TLR2, TLR6 and TLR9 in B-1 cells, pointing to impaired innate immune sensing despite ongoing immune activation and inflammatory responses (231). Taken together, these observations suggest that aberrant TLR signaling, particularly through the TLR4 and TLR2/6 pathways, plays a central role in the immune dysregulation and chronic inflammatory state characteristic of OSA.

### 8.4 NLRP3 inflammasome

The NLRP3 inflammasome is a central regulator of innate immune responses that promotes inflammation through caspase-1 activation and the subsequent maturation and secretion of IL-1β and IL-18. Accumulating evidence suggests that NLRP3 signaling is activated in obstructive sleep apnea (OSA), where it may contribute to IH-induced inflammation, oxidative stress and tissue damage (247, 261–264). Clinical evidence indicates that NLRP3 inflammasome activity is elevated in circulating monocytes from patients with severe OSA. Moreover, IH potentiates the effects of oxidized low-density lipoprotein (oxLDL) and OSA-derived plasma on NLRP3 activation and IL-1β secretion, highlighting a synergistic interplay between hypoxic stress and inflammatory mediators in promoting inflammasome signaling (247, 261–264). However, direct biomarkers confirming *in vivo* inflammasome activation in human tissues remain scarce.

Experimental studies provide mechanistic support for the involvement of NLRP3 inflammasome signaling in OSA pathogenesis. Genetic deletion or pharmacological inhibition of NLRP3 significantly reduces IH-induced inflammation, oxidative stress and tissue damage in both the brain and cardiovascular system, underscoring the importance of this pathway in the development of OSA-associated complications (247, 264). Consistent with these findings, IH promotes activation of the pulmonary NLRP3 inflammasome, resulting in elevated expression of NLRP3 and its downstream effectors, including IL-1β, IL-18 and caspase-1 in lung tissue. The marked attenuation of these inflammatory responses in NLRP3-deficient mice further underscores the pivotal role of NLRP3 signaling in the pathogenesis of IH-induced pulmonary inflammation (244). Because most mechanistic evidence derives from animals and *in vitro* models, these findings cannot be directly extrapolated to human OSA. Collectively, NLRP3 inflammasome activation represents a potential mediator of OSA associated inflammation and complications. However, NLRP3r signaling related immune biomarkers remain at exploratory stage, lack external validation, and are not currently reported for OSA subtyping or treatment-response prediction in routine clinical practice. **Table 1** provides an overview of the principal cytokines involved in innate, adaptive and humoral immune responses, emphasizing their biological functions, signaling mechanisms, and relevance to OSA pathogenesis.

**Table 1:** Major immune and inflammatory markers associated with Obstructive Sleep Apnea (OSA).

Markers	Change	Primary source	Physiological function	Pathway/mechanism	Clinical significance/impact in OSA	Evidence source	References
IL-1β or α	↑ Increased	Monocytes and macrophages	Inflammasome activation	NLRP3 inflammasome activation	Sleep fragmentation, cardiovascular risk and endothelial dysfunction	Human cross-sectional; Human intervention; Animal; *In vitro*	(60, 63, 122, 159, 164, 173, 177, 180, 189, 190, 209, 244, 265–271)
IL-2	↓ Decreased	Activated T cells	T-cell proliferation	Chronic inflammatory suppression	Adaptive immune dysfunction and impaired immune regulation	Human cross-sectional; Human intervention	(63, 118, 196, 266, 272)
IL-4/Th2	Variable/Often decreased	Th2 cells	Humoral immunity	Th1/Th17 skewing	Th1 dominance and airway inflammation	Human cross-sectional; Human intervention; Animal	(63, 112, 122, 126, 182, 196, 272, 273)
IL-6	↑ Predominantly Increased (few studies reported variable results of decreased or unchanged)	Monocytes and adipocytes	Systemic inflammation	Hypoxia and oxidative stress	Insulin resistance, obesity, ASCVD and severe OSA	Human cross-sectional; Human intervention; Animal; *In vitro*	(39, 60, 62–64, 69, 103, 110, 122, 159, 167, 175, 177, 181, 189, 190, 196, 208, 209, 230, 238, 265–268, 270, 272, 274–277)
IL-8 (CXCL8)	↑ Increased	Macrophages and epithelial cells	Neutrophil recruitment	NF-κB activation	Neutrophilic airway inflammation and OSA severity	Human cross-sectional; Animal; *In vitro*	(39, 62–69, 227)
IL-10	↓ Decreased	Tregs and macrophages	Anti-inflammatory cytokine	Treg dysfunction	Chronic inflammation and immune imbalance	Human cross-sectional; Human intervention; Animal	(69, 103, 112, 122, 173, 174, 189, 229, 231, 267)
IL-12	↑ Increased	Macrophages and dendritic cells	Th1 activation	Hypoxia-mediated immune activation	Systemic inflammatory progression	Human cross-sectional	(63, 238, 273)
IL-17A	↑ Increased	Th17 cells	Neutrophilic inflammation	Th17/Treg imbalance	Airway inflammation and endothelial dysfunction	Human cross-sectional; Human intervention; Animal	(63, 122, 126, 174, 232, 238, 267, 278)
IL-18	↑ Increased	Monocytes and macrophages	Pro-inflammatory cytokine	Inflammasome activation	Metabolic syndrome and cardiovascular risk	Human cross-sectional; Animal	(63, 238, 240, 244, 266)
IL-23	↑ Increased	Dendritic cells and macrophages	Th17 cell differentiation and maintenance	IL-23/Th17 axis activation → ↑ IL-17, neutrophilic inflammation and NF-κB signaling	Chronic inflammation, oxidative stress, airway inflammation and cardiovascular/metabolic complications	Human cross-sectional; Human intervention	(238, 267)
IL-35	↑ Increased	Regulatory T cells	Anti-inflammatory regulation	Compensatory immune response	Protective anti-inflammatory response	Human cross-sectional	(250, 279)
IL-37	↑ Increased	Monocytes	Anti-inflammatory effects	Inflammatory compensation	Potential protective biomarker	Human cross-sectional	(250, 279)
TNF-α	↑ Increased predominantly (some studies report variable results)	Macrophages and T cells	Inflammatory signaling	NF-κB activation	Daytime sleepiness, metabolic dysfunction and atherosclerosis	Human cross-sectional; Human intervention; Animal; *In vitro*	(39, 62, 64, 69, 112, 122, 166, 177, 180–182, 208, 267, 268, 270, 271, 276–278, 280, 281)
IFN-γ	↑ Predominantly Increased/Variable	Th1 cells and NK cells	Macrophage activation	Th1 polarization	Systemic inflammation and insulin resistance	Human cross-sectional	(60, 282)
TGF-β	Variable	Tregs and fibroblasts	Fibrosis and repair	Chronic tissue injury	Fibrosis, airway and vascular remodeling	Human cross-sectional; Animal	(122, 174, 180, 271, 273)
CRP	↑ Increased	Liver	Acute phase response	IL-6 stimulation	Predicts OSA severity and cardiovascular events	Human cross-sectional; Human intervention	(39, 59, 64, 69, 122, 267, 269, 274–276, 280, 283–285)
MCP-1/CCL2	↑ Increased	Endothelial cells	Monocyte recruitment	Inflammatory cytokine signaling	Atherosclerosis and vascular inflammation	Human cross-sectional; Human intervention	(65, 66, 272)
ICAM-1	↑ Increased	Endothelial cells	Leukocyte adhesion	Endothelial activation	Endothelial dysfunction and vascular risk	Human cross-sectional; *In vitro*	(39, 65, 67, 68, 276, 286–289)
VCAM-1	↑ Increased	Endothelial cells	Endothelial activation	Oxidative stress	Atherogenesis and vascular injury	Human cross-sectional; *In vitro*	(39, 67, 287–289)
L-selectin	↑ Increased	Leukocytes	Leukocyte rolling	Immune activation	Systemic inflammatory activation	Human cross-sectional	(39, 166, 289)
VEGF	↑ Increased	Endothelial cells	Angiogenesis	HIF-1α activation	Vascular remodeling and endothelial dysfunction	Human cross-sectional; Human intervention; *In vitro*	(33, 166, 272)
HIF-1α	↑ Increased	Hypoxia-responsive factor	Hypoxia adaptation	Intermittent hypoxia	Hypoxic inflammation and oxidative stress	Human cross-sectional; Animal	(64, 126, 166, 283, 290)
NLR and neutrophils	↑ Increased	CBC-derived	Inflammation marker	Neutrophilia and lymphopenia	Predictor of disease severity and CVD risk	Human cross-sectional; Animal; *In vitro*	(53, 94, 97, 98, 105, 227)
B- cells/CD80 CD86	↑ Increased predominantly/Variable					Animal	(114, 120, 121, 231)
CD4^+^ T Cells	↑Decreased generally/Variable	Helper T cells	Adaptive immunity	Intermittent hypoxia	Immune activation and chronic inflammation	Human cross-sectional; Animal; *In vitro*	(60, 96, 122, 124, 268, 282)
CD3+ T cells	↓ Decreased generally	Mature T lymphocytes	Cellular immune defense	Intermittent hypoxia–induced immune alteration	Reflects systemic immune dysfunction and inflammatory stress in OSA	Human cross-sectional; *In vitro*	(268)
CD8+ T cells	↑ Increased/activated/ variable	Cytotoxic T lymphocytes	Kill infected or damaged cells	Intermittent hypoxia–driven cytotoxic and inflammatory activation	Contribute to systemic inflammation, endothelial injury and immune dysregulation in OSA	Human cross-sectional; *In vitro*	(268)
CD40 cells	↑ Increased	APCs, endothelial cells	Costimulatory signaling	NF-κB activation	Atherosclerotic and vascular risk	Human cross-sectional	(112)
NKT Cells	Variable/often decreased ↓	Natural killer T cells	Immune surveillance	Hypoxia-related dysregulation	Immune dysfunction and cancer susceptibility	Human cross-sectional; Human intervention	(58, 117, 118, 282)
CD16^+^/CD14^+^/ Monocytes	↑ Increased	Pro-inflammatory monocytes	Cytokine production	Hypoxia activation	Vascular inflammation and endothelial injury	Human cross-sectional; *In vitro*	(101, 282, 291, 292)
Monocytes-Macrophages	↑ Increased	Pro-inflammatory monocytes	Cytokine production	Hypoxia activation	Inflammation and endothelial injury	Animal	(97, 103, 105, 108, 109)
Monocyte–T Cell Complexes	↑ Increased	Monocytes and T cells	Immune interaction	Systemic inflammation	Immune activation burden	Human cross-sectional; Animal	(96, 101, 108)
Tregs	↑ Decreased predominantly/Variable	CD4^+^CD25^+^FOXP3^+^ T lymphocytes	Immune suppression, maintenance of immune tolerance and inhibition of excessive inflammation	Intermittent hypoxia and oxidative stress induced Th17/Treg imbalance, reduce FOXP3 expression and impair anti-inflammatory signaling (IL-10, TGF-β)	Associated with chronic systemic inflammation, endothelial dysfunction, atherosclerosis, immune dysregulation and increased OSA severity	Human cross-sectional; Animal	(122, 126, 174, 282, 293)
Foxp3	↓ Decreased	Regulatory T cells (Tregs)	Master transcription factor regulating Treg development, immune tolerance and anti-inflammatory responses	IH and oxidative stress suppress FOXP3 expression, promoting Th17/Treg imbalance and inflammatory activation	Associated with reduced immune regulation, chronic systemic inflammation, endothelial dysfunction and increased OSA severity	Human cross-sectional	(174)
Th17 cells	↑ Increased	CD4^+^ T helper lymphocytes	Pro-inflammatory adaptive immunity, neutrophil recruitment and mucosal defense	IH, oxidative stress and inflammatory cytokines (IL-6, IL-1β, IL-23) promote Th17 differentiation via RORγt activation and thus Th17/Treg imbalance	Associated with chronic systemic inflammation, endothelial dysfunction, airway inflammation, cardiovascular risk	Human cross-sectional	(122, 174, 232, 278)
Th2/IL-4	Variable/Often Decreased ↓	Th2 CD4^+^ T cells, mast cells, basophils	Humoral immunity, B-cell activation, class switching, anti-inflammatory responses	Th1/Th17 skewing, intermittent hypoxia–induced immune imbalance	Reduced Th2/IL-4 activity may contribute to Th1 dominance, chronic inflammation and airway inflammation in OSA	Human cross-sectional; Human intervention; Animal	(63, 112, 122, 126, 196, 272, 273)
Th1/IFN-γ	↑ Increased Predominantly/Variable	Th1 CD4^+^ T cells, CD8^+^ T cells, NK cells, NKT cells	Cell-mediated immunity, macrophage activation, intracellular pathogen defense	Intermittent hypoxia, oxidative stress and Th1 polarization; Th1/Th2 imbalance	Promotes systemic inflammation, macrophage activation, endothelial dysfunction, insulin resistance and cardiovascular complications in OSA	Human cross-sectional; Animal	(60, 63, 122, 126, 278, 282)
IgA	↑ Increased Predominantly/Variable	Plasma cells in mucosal tissues and lamina propria B cells	Mucosal immune defense and pathogen exclusion	Mucosal B-cell activation and class-switch recombination	May influence upper-airway inflammation, epithelial barrier function and microbiota interactions in OSA	Human cross-sectional; Human intervention	(79, 128, 129, 135)
IgG	Variable: transiently increased or decreased	Plasma cells, class-switched B cells	Neutralization, opsonization, complement activation, immunological memory	Germinal center reactions, class-switch recombination, plasma-cell differentiation	Altered adaptive humoral immunity and impaired vaccine responsiveness in OSA/SF	Human cross-sectional; Human intervention; Animal; *In vitro*	(128–130, 132, 133, 135)
IgM	Predominantly preserved or increased	B-1 cells, naïve B cells, short-lived plasma cells	Early humoral defense and complement activation	T-independent responses and early B-cell activation	Reflects innate-like humoral activation and systemic inflammation in OSA	Human cross-sectional; Human intervention; Animal	(128, 129, 133–135, 138)
Complement C3	↑ Increased Predominantly	Hepatocytes, macrophages, monocytes	Opsonization, complement activation, inflammatory amplification	Classical, lectin and alternative complement pathways	May contribute to endothelial dysfunction and chronic inflammation in OSA	Human cross-sectional; Human intervention; Animal	(128, 134, 135, 139, 140)
Complement C4	↑ Increased Predominantly	Hepatocytes, monocytes, macrophages	Classical and lectin pathway activation	Complement cascade activation	Supports systemic inflammatory responses and immune dysregulation in OSA	Human cross-sectional; Human intervention	(128, 135, 139, 140)
PD-1 (T cells)	↑ Increased	Activated T cells	Immune checkpoint inhibition	Chronic immune stimulation	T-cell exhaustion and dysfunction	Human cross-sectional; Animal; *In vitro*	(101, 254)
PD-L1 (Monocytes)	↑ Increased	Macrophages/Monocytes	Immune inhibitory signaling	Inflammatory activation	Altered immune cross-talk	Human cross-sectional; Animal; *In vitro*	(101, 254)
Surfactant Protein-D	↓ Decreased	Pulmonary epithelial cells	Innate lung defense	Airway injury	Pulmonary epithelial dysfunction	Human cross-sectional	(64)
TLR2/6	↑ Increased	Macrophages, monocytes, epithelial cells	Recognition of microbial lipoproteins and innate immune activation	Activates MyD88 → NF-κB signaling → ↑ IL-1β, IL-6, TNF-α	Promotes systemic inflammation, gut-derived immune activation and endothelial dysfunction	Human cross-sectional; Animal	(231, 259, 260)
TLR4	↑ Increased	Monocytes/macrophages, adipose tissue, airway epithelium	Detection of LPS/endotoxins and mediates inflammatory response	LPS–TLR4 activation → NF-κB and inflammasome signaling → cytokines release	Associated with IH–induced inflammation, insulin resistance, oxidative stress and cardiovascular risk	Human cross-sectional; Animal	(38, 208, 255–258)
NF-κB	↑ Increased	Immune cells	Inflammatory transcription	Oxidative stress activation	Chronic systemic inflammation	Human cross-sectional; Animal; *In vitro*	(38, 64, 126, 190, 196, 208, 209, 257, 258, 291)
Perforin-positive γδ-T Cells	↓ Decreased	γδ T lymphocytes	Cytotoxic defense	Inflammatory impairment	Reduced immune surveillance	Human cross-sectional	(123)

^a^Reported magnitude or direction varies across studies, reflecting differences in adiposity, age, OSA severity, comorbidities, sampling, assay methods and experimental designs.

Evidence source labels identify the primary designs represented by the cited studies, a row may contain more than one evidence type.

↑, increased; ↓, decreased; variable represents inconsistent direction or magnitude across studies including increased, decreased, or unchanged findings.

AHI, apnea–hypopnea index; CBC, complete blood count; CCL2, C-C motif chemokine ligand 2; CD, cluster of differentiation; CRP, C-reactive protein; HIF-1α, hypoxia-inducible factor 1-alpha; ICAM-1, intercellular adhesion molecule 1; IFN-γ, interferon-gamma; Ig, immunoglobulin; IL, interleukin; MCP-1, monocyte chemoattractant protein 1; NF-κB, nuclear factor kappa B; NK, natural killer; NKT, natural killer T; NLR, neutrophil-to-lymphocyte ratio; OSA, obstructive sleep apnea; PD-1, programmed cell death protein 1; PD-L1, programmed death ligand 1; SCFA, short-chain fatty acid; TGF-β, transforming growth factor-beta; Th, T-helper; TLR, Toll-like receptor; TNF-α, tumor necrosis factor-alpha; Treg, regulatory T cell; VCAM-1, vascular cell adhesion molecule 1; VEGF, vascular endothelial growth factor.

## 9 Gut microbiota and immune dysregulation in OSA

IH, a defining physiological characteristic of OSA, is increasingly recognized as a potential contributor to gut microbial dysbiosis and immune dysregulation. Accumulating experimental and clinical evidence suggests that OSA-induced alterations in gut microbial composition promote systemic inflammation by influencing innate and adaptive immune pathways. These alterations impair intestinal barrier function and alter the production of immunomodulatory microbial metabolites (294). However, human observational studies cannot establish whether microbial alterations are consequences of IH and sleep disruption or independent drivers of systemic inflammation. The gut microbiota plays a central role in maintaining immune homeostasis, in part through regulation of the Th17/Treg axis (295, 296). Distinct commensal bacterial taxa promote specific immune phenotypes; segmented filamentous bacteria drive Th17 cell differentiation, whereas Clostridia species enhance the expansion of Foxp3^+^ regulatory T cells (Tregs) through the generation of short-chain fatty acids (SCFAs), particularly butyrate and propionate. These microbial-immune interactions are critical for preserving mucosal immune balance and limiting excessive inflammatory responses (295, 296). Short-chain fatty acids (SCFAs) possess potent immunomodulatory properties and exert anti-inflammatory effects through histone deacetylase (HDAC) inhibition and G protein-coupled receptor-mediated signaling. These mechanisms promote the differentiation and expansion of regulatory T cells (Tregs) while suppressing proinflammatory immune activation (297). Dysbiosis-associated depletion of SCFA-producing bacteria may therefore favor expansion of Th17 and Th1 inflammatory responses and potentially contribute to chronic systemic inflammation (298). In addition to shaping adaptive immune responses, the gut microbiota modulates innate immune signaling through persistent interactions with pattern-recognition receptors. Dysbiosis marked by the enrichment of Gram-negative bacteria may increase lipopolysaccharide (LPS) exposure, leading to activation of the TLR4/NF-κB signaling pathway and enhanced production of proinflammatory cytokines such as TNF-α, IL-1β, and IL-6 (299). These pathways are of particular relevance to OSA, in which IH could interact with microbiota-derived inflammatory stimuli to exacerbate systemic immune activation and chronic inflammation. Nevertheless, this proposed interaction has been established principally in experimental models and requires confirmation in patients with OSA.

Patients with OSA exhibit reduced levels of SCFA-producing bacteria and an increased abundance of potentially pathogenic taxa. These microbial alterations are associated with elevated IL-6 concentrations, despite no significant differences in TNF-α levels, supporting an association, rather than a proven causal relationship. Notably, the Ruminococcus enterotype is associated with a higher risk of OSA, whereas Lactobacillus abundance shows an inverse relationship with homocysteine levels, suggesting a potential role for the gut microbiota in modulating inflammatory and metabolic pathways in OSA. These associations suggest that the gut microbiota may be related to inflammatory and metabolic pathways in OSA, but they do not demonstrate that microbial changes cause these abnormalities. Guo et al. performed a clinical cohort study involving 113 adults, including 16 healthy controls and 97 OSA patients stratified into mild, moderate and severe disease groups (269). Fecal 16S rRNA sequencing demonstrated reduced microbial diversity and depletion of SCFA-producing genera, including Faecalibacterium and Gemmiger, with increasing OSA severity. Elevated IL-1β and CRP levels were also observed in OSA patients. Importantly, hypoxic burden was strongly associated with microbial dysbiosis, linking IH to gut microbiota alterations and systemic inflammation, indicating a relationship among IH, microbial alterations and systemic inflammation without establishing the direction of causality. A subsequent study of 37 adults used metagenomic sequencing and serum cytokine analysis to further characterize these relationship (278). Reduced abundance of Faecalibacterium prausnitzii and Bifidobacterium adolescent is in OSA was associated with elevated IL-17A and TNF-α levels. Both taxa were inversely correlated with IL-17A and disease severity, supporting a relationship between gut dysbiosis and Th17-driven inflammation. However, the small sample size and observational design prevent conclusions regarding whether dysbiosis causes inflammation or develops secondary to OSA-related physiological disturbances.

Disruption of intestinal barrier integrity represents a major mechanistic link between OSA-induced gut dysbiosis and immune dysfunction. Experimental animal studies provide mechanistic support for the associations observed in human OSA cohorts. IH exposures *in vivo* reduce the expression of key epithelial tight-junction proteins, including zonula occludens-1 (ZO-1), occludin and claudins, resulting in increased intestinal permeability. This compromised barrier function facilitates the translocation of bacterial products, such as lipopolysaccharide (LPS), into the systemic circulation, thereby promoting systemic inflammation and immune dysregulation (300). Circulating LPS activates Toll-like receptor 4 (TLR4) signaling pathways in monocytes, macrophages, endothelial cells and hepatocytes, leading to NF-κB activation and subsequent production of pro-inflammatory cytokines, including TNF-α, IL-1β, and IL-6. Concurrently, LPS promotes activation of the NLRP3 inflammasome, further amplifying inflammatory responses through the maturation and release of IL-1β and IL-18 (270, 301). These mechanisms have been demonstrated primarily in experimental models and cannot yet be assumed to occur to the same extent in human OSA. Evidence supporting a potentially causal role of the gut microbiota has emerged from experimental studies, including the fecal microbiota transplantation (FMT) study (300). Gut microbiota obtained from patients with OSA, with or without hypertension, were transplanted into germ-free mice randomized to receive fecal microbiota from healthy controls, OSA patients, OSA patients with pre-hypertension, or OSA patients with hypertension. Recipient mice exhibited elevated systolic blood pressure, vascular injury and increased circulating lipopolysaccharide (LPS) levels. They also exhibited enhanced expression of TLR4 and phosphorylated NF-κB in aortic tissues (300). These results show that OSA-associated microbial communities can transfer inflammatory and metabolic phenotypes under controlled experimental conditions in mice. However, they do not establish that gut dysbiosis causes systemic inflammation or cardiovascular disease in patients with OSA. Additional studies from our group have clearly demonstrated the unique roles potentially played by changes in gut microbiome in end-organ morbidity as exerted by the presence of perturbations that characterize OSA (302, 303). These animal findings support a plausible causal hypothesis but do not prove that OSA-associated dysbiosis is an independent driver of systemic inflammation or cardiovascular pathology in humans.

In addition, depletion of short-chain fatty acids (SCFAs) may further exacerbate immune dysregulation in OSA. SCFAs, primarily produced by commensal bacteria such as *Faecalibacterium prausnitzii*, *Roseburia* spp. and members of the *Clostridia* class, play a critical role in maintaining epithelial barrier integrity, suppressing NF-κB signaling, promoting Foxp3^+^ regulatory T-cell differentiation, and inhibiting dendritic cell maturation. Consequently, reduced SCFA production may enhance intestinal permeability and perpetuate the pro-inflammatory immune environment associated with OSA (304). Consequently, the loss of SCFA-producing bacteria in OSA may therefore weaken anti-inflammatory regulatory mechanisms, leading to enhanced systemic inflammation and a shift in the Th17/Treg balance toward a pro-inflammatory immune state (304). This proposed pathway remains mechanistically plausible but has not been causally confirmed in human OSA.

Several murine studies have demonstrated that IH and sleep fragmentation (SF), the two hallmark features of OSA, directly alter gut microbial composition and contribute to immune dysregulation. In mice, six weeks of IH exposure sufficient to model clinically relevant OSA resulted in significant alterations in fecal microbial diversity and community structure relative to normoxic controls (305). Specifically, IH increased the relative abundance of *Firmicutes* while decreasing *Bacteroidetes* and *Proteobacteria*. These alterations coincided with measurable hypoxia–reoxygenation fluctuations within the colonic lumen, indicating that intestinal microbes are directly exposed to oxygen cycling and supporting a potential mechanism through which IH could alter the gut microbiota (305). Similarly, longitudinal exposure to IH and hypercapnia induced significant alterations in the gut microbiome and metabolome. These affect more than 10% of microbial species and over 22% of metabolites detected, including bile acids, fatty acids and enterolignans. A marked depletion of *Clostridia* species, a major source of SCFAs and an important inducer of regulatory T cells (Tregs), was consistent with the possibility of impaired immune tolerance, disrupted gut immune homeostasis and increased susceptibility to inflammation (293). CIH induced enrichment of *Bacteroides* and *Desulfovibrionaceae* and depletion of *Bifidobacterium*, changes that were associated with increased systemic inflammation and immune activation (306). Sleep fragmentation (SF) also promotes gut microbiota–associated immune dysregulation in experimental models. In mice, SF reduced expression of key immune mediators in mesenteric lymph nodes while increasing cortical IL-33 expression, indicating concurrent mucosal immune suppression and neuroinflammatory activation and suggesting possible involvement of the gut–brain axis (307). Consistent with these findings, sleep fragmentation–induced intestinal inflammation and barrier dysfunction are thought to promote the translocation of lipopolysaccharide (LPS) into the circulation, thereby activating innate immune signaling pathways and driving the recruitment of inflammatory myeloid cells into atherosclerotic lesions (98). Because these findings were derived from animal sleep-fragmentation or IH models, they cannot be directly equated with the complex clinical condition of human OSA.

In summary, OSA-associated IH may promote gut dysbiosis, intestinal barrier dysfunction, and LPS-mediated inflammation, potentially activating TLR4/NF-κB and NLRP3 signaling and disrupting the Th17/Treg balance. Collectively, these findings suggest that gut dysbiosis may contribute to systemic immune dysfunction in OSA, rather than representing an established causal driver. However, these mechanistic links are supported largely by experimental animal studies and require validation in well-designed human interventional studies. A proposed mechanistic pathway is illustrated in **Figure 3**.

**Figure 3:**
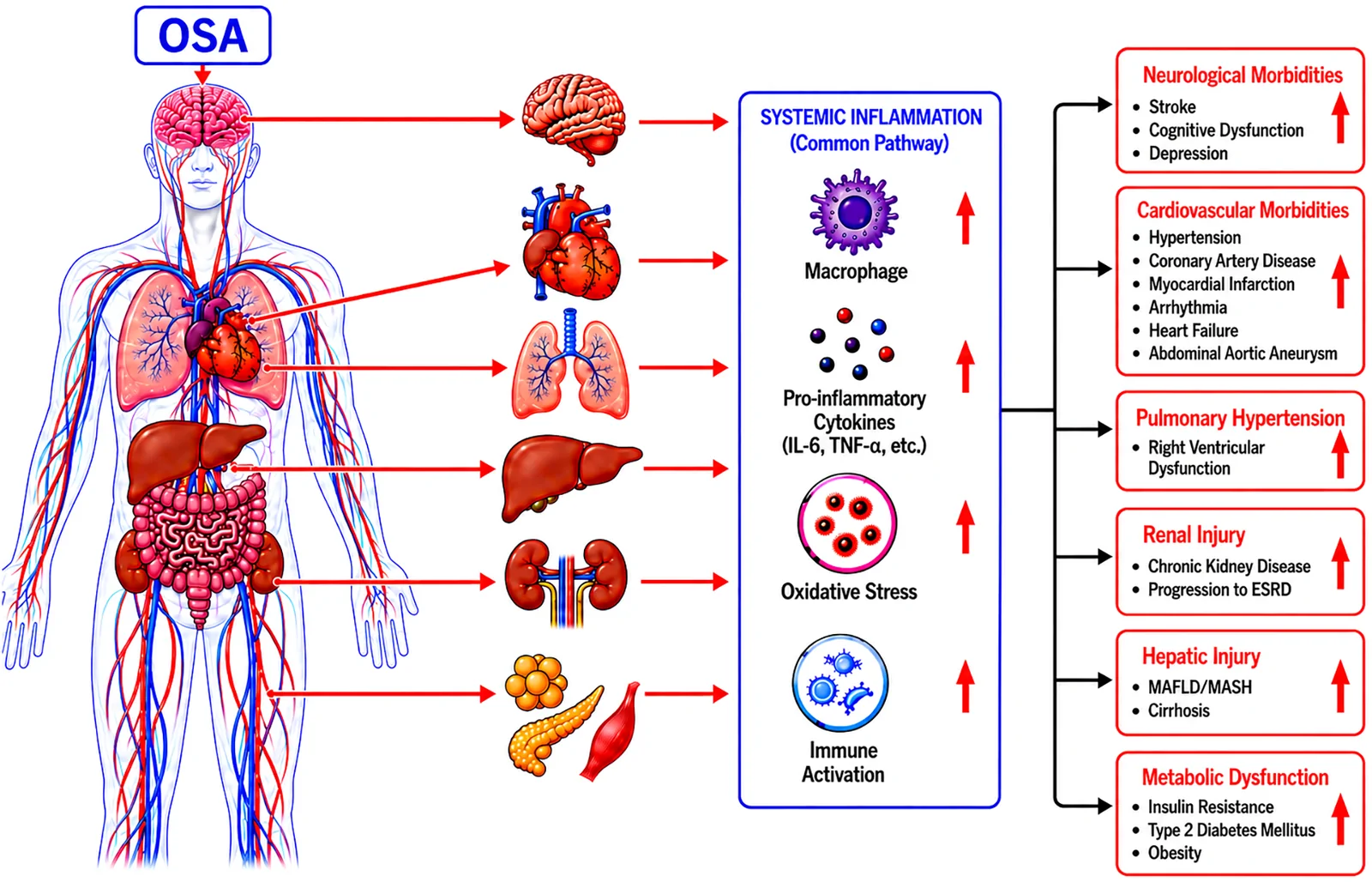
Proposed mechanism linking obstructive sleep apnea (OSA) to systemic inflammation through gut microbiota dysbiosis and intestinal barrier dysfunction. OSA-associated factors, including intermittent hypoxia, sleep fragmentation, sympathetic activation, oxidative stress, metabolic disturbances and obesity-related dietary influences, promote alterations in gut microbial composition. These changes are characterized by a reduction in beneficial short-chain fatty acid (SCFA)-producing bacteria (e.g., *Faecalibacterium*, *Roseburia*, *Akkermansia*, and *Bifidobacterium*) and an increase in endotoxin-producing microorganisms. The resulting decrease in SCFA production and increase in lipopolysaccharide (LPS) levels contribute to disruption of intestinal barrier integrity, including reduced expression of tight junction proteins (occludin, claudin and ZO-1), mucus layer impairment and epithelial damage, leading to increased intestinal permeability (leaky gut). Translocation of LPS and other microbial products into the circulation activates pattern recognition receptors, particularly Toll-like receptor 4 (TLR4), triggering MyD88-dependent signaling, NF-κB activation and inflammasome pathways (NLRP3). These processes promote activation of innate and adaptive immune cells, including macrophages, dendritic cells, Th1 and Th17 cells, while reducing regulatory T-cell (Treg) activity, resulting in cytokine release, immune dysregulation and systemic inflammation. Human studies primarily demonstrate associations among OSA severity, hypoxic burden, gut microbial alterations, and circulating inflammatory markers, whereas evidence for intestinal barrier disruption and downstream mechanistic pathways is derived largely from *in vivo* and *in vitro* studies. This figure schematically summarizes the proposed mechanisms linking OSA to gut dysbiosis and systemic inflammation; however, several of these pathways remain to be validated in human OSA cohorts.SCFAs, short-chain fatty acids; LPS, lipopolysaccharide; TLR4, Toll-like receptor 4; NF-κB, nuclear factor kappa B; NLRP3, NOD-like receptor family pyrin domain-containing 3. SCFAs=short-chain fatty acids; LPS=lipopolysaccharide; TLR4=Toll-like receptor 4; MyD88=myeloid differentiation primary response 88; NF-κB=nuclear factor kappa B; NLRP3=NOD-like receptor family pyrin domain containing 3; Th1=T helper 1; Th17=T helper 17; Treg= regulatory T cell; ZO-1=zonula occludens-1.

The immune system and the central nervous system (CNS) are two highly integrated systems that continuously detect environmental stimuli. They generate adaptive responses and retain information through memory mechanisms that enable more efficient responses to future challenges. Close bidirectional communication between these systems is essential for maintaining homeostasis and adapting to environmental change. For example, acute mental or physical stressors that primarily activate CNS-regulated pathways, including the autonomic nervous system (ANS) and the hypothalamic–pituitary–adrenal (HPA) axis, can also trigger inflammatory immune responses (308). Conversely, stimuli that primarily engage the immune system, such as antigenic challenges, can also lead to alterations in neurobehavior (309). This phenomenon supports the concept that the immune system functions as a “sixth sense,” capable of detecting environmental stimuli and communicating this information to the brain (310). Leukocyte trafficking to the brain and spinal cord, along with soluble mediators and nerve fibers, establishes important communication pathways between the immune system and the central nervous system. In addition, sympathetic, peptidergic and sensory nerve fibers innervate both primary and secondary lymphoid organs, further facilitating bidirectional neuroimmune interactions (311). Matching receptors expressed on immune cells can identify a variety of neurotransmitters and neuropeptides released from efferent neurons, including catecholamines and neuropeptide Y. In addition, leukocytes themselves are also capable of producing and releasing these neuronal messengers (312). The cholinergic innervation of immune organs is still a matter of debate (313), yet a non-neuronal cholinergic system in T cells can locally provide acetylcholine release in the periphery (314). Neurotransmitters and neuropeptides generated by local nerve terminals can be directly detected by tissue-resident leukocytes such as macrophages in addition to lymphoid organs (Ordovas-Montanes et al., 2015). In contrast to the rapid communication mediated through nerve fibers, the neuroendocrine axis provides an additional, relatively slower mechanism for regulating the immune system. Leukocytes have the ability to detect and produce a wide range of neuroendocrine mediators at the hypothalamic, pituitary and glandular levels. These mediators include corticotropin-releasing hormone, adrenocorticotropic hormone, cortisol, growth hormone-releasing hormone, growth hormone (GH), insulin-like growth factor-1 (IGF-1), dopamine and prolactin. They can even create their own, local neuroendocrine axis in an autocrine and paracrine manner (312). Leukocytes can communicate with the brain through afferent nerve fibers by synthetizing and releasing neurotransmitters, neuropeptides, and hormones. Additional mediators of immune-to-brain signaling include immunopeptides such as cytokines and chemokines, which are produced by immune cells and act on neurons, astrocytes and microglia within the peripheral nervous system (PNS) and central nervous system (CNS) through their corresponding receptors. These substances, along with other inflammatory agents such as PGs, PAMPs and DAMPs can enter the PNS or CNS through leaky locations of circumventricular organs or transporter systems that traverse the blood-brain barrier. Immune mediators that stimulate vagal and other neural afferents, cerebral endothelial and perivascular cells, or promote the directed migration of leukocytes to the brain represent alternative pathways of immune-to-brain signaling (315, 316). All these pathways may induce microglial activation and neuroinflammation in response to peripheral inflammation, thereby initiating CNS responses including fever or sickness behavior (317). Autoreactive T and B cells add an additional level of complexity, as they are capable of regulating inflammatory processes within the brain (318) and might represent a reflection of “our self,” the “immunculus,” to restore homeostasis of tissues and organs (319). Neurons, glia cells and nerve fibers themselves can synthesize cytokines and chemokines, demonstrating a further pathway of brain-to-immune signaling (320). Overall, these findings provide corroborative evidence indicating that the CNS and immune system communicate within and across systems through a shared biochemical language. Thus, the classification of neurotransmitters, neuropeptides, hormones and immune mediators largely reflects the order of their discovery rather than their exclusive association with a particular system. The experimental setting used to investigate the neuro-immune interactions of interest determines the final outcomes of these brain-to-immune or immune-to-brain signaling pathways. For instance, depending on whether it occurs before or after the immunological challenge, activation of the classical HPA and ANS stress axes can either promote or inhibit inflammatory and adaptive immune responses in both human and animal models (321). As a result, many hormones, neurotransmitters and neuropeptides cannot generally be categorized as immunosuppressive or immune-supportive, rather their actions depend on the specific context. Overall, the evidence indicates that the immune response, often characterized by the four Rs—*recognition, reaction, regulation and resolution*, is fine-tuned through bidirectional brain-immune interactions. The effects of the stress axis including priming immune cells and inflammation as well as mobilizing energy substrates to support the immune system, facilitate recognition and response during the early immediate pro-inflammatory phase (322, 323). Subsequent counter-inflammatory actions that limit the inflammatory process in both space and time are referred to as regulation and resolution. At this stage, the immune system is regulated at two levels. At one level, the immune system employs intrinsic control mechanisms and pro-resolving mediators to regulate itself including regulatory T cells (Tregs), indoleamine 2,3-dioxygenase, M2 macrophages, IL-10, resolution-associated molecular patterns (RAMPs) and specialized pro-resolving lipid mediators (324). On the other hand, the CNS exerts superordinate extrinsic control through its efferent pathways such as the HPA and ANS stress axes. This balanced and coordinated regulation ensures a highly effective and transient local immune response with low energy expenditure and minimal collateral damage (325).

Experimental evidence indicates that IH induces neuroimmune alterations involving both innate and adaptive immune pathways. Acute IH increases hippocampal expression of IL-1β and RANTES/CCL5, while chronic IH promotes microglial activation and expansion, consistent with progressive neuroinflammation (189). Chronic IH also enhanced IL-6 and IL-10 responses following LPS challenge indicating altered innate immune responsiveness. Chronic IH upregulated NLRP3, IL-1β, IL-18 and caspase-1 expression in key brain regions, whereas these responses were markedly attenuated in NLRP3-deficient mice, highlighting a critical role for NLRP3-mediated neuroinflammation (244). SF also alters neuroimmune homeostasis. Chronic SF decreases hypothalamic hypocretin expression, promoting CSF1-mediated myelopoiesis, expansion of inflammatory monocytes and accelerated atherosclerotic disease (97). SF increased brain CD11b expression and peripheral myelopoiesis in Apoe^−^/^−^ mice, providing further evidence that sleep disruption promotes neuroinflammation (105). SF also affects adaptive immunity, increasing CD4^+^ T-cell infiltration into the spinal cord and indicating altered immune-cell trafficking within the CNS (124). Acute sleep deprivation increased B-cell infiltration into the brain, meninges and choroid plexus. It was associated with increased CXCR5 and CXCL13 expression, supporting a role for sleep loss in neuroimmune remodeling (120). Sleep loss also disrupts B-cell homeostasis, reducing B-lymphocyte populations in the bone marrow and spleen (114). Chronic sleep restriction shifted B-1 cells toward an activated myeloid-like phenotype and increased expression of the co-stimulatory molecules CD80 and CD86, indicating enhanced cellular activation (231).

Bidirectional interactions between neuroimmune pathways and sleep regulation contribute to reduced NREM and slow-wave sleep and increased production of pro-inflammatory cytokines, including IL-1β and TNF-α, leading to neuroendocrine and systemic dysfunction (326), while at the same time bidirectional dysregulation of the hypothalamic–pituitary–adrenal (HPA) axis and sleep leads to disturbances in stress hormones (cortisol, CRH, ACTH), collectively worsening sleep quality and contributing to systemic and neuroendocrine dysfunction (327) as shown in **Figure 4**.

**Figure 4:**
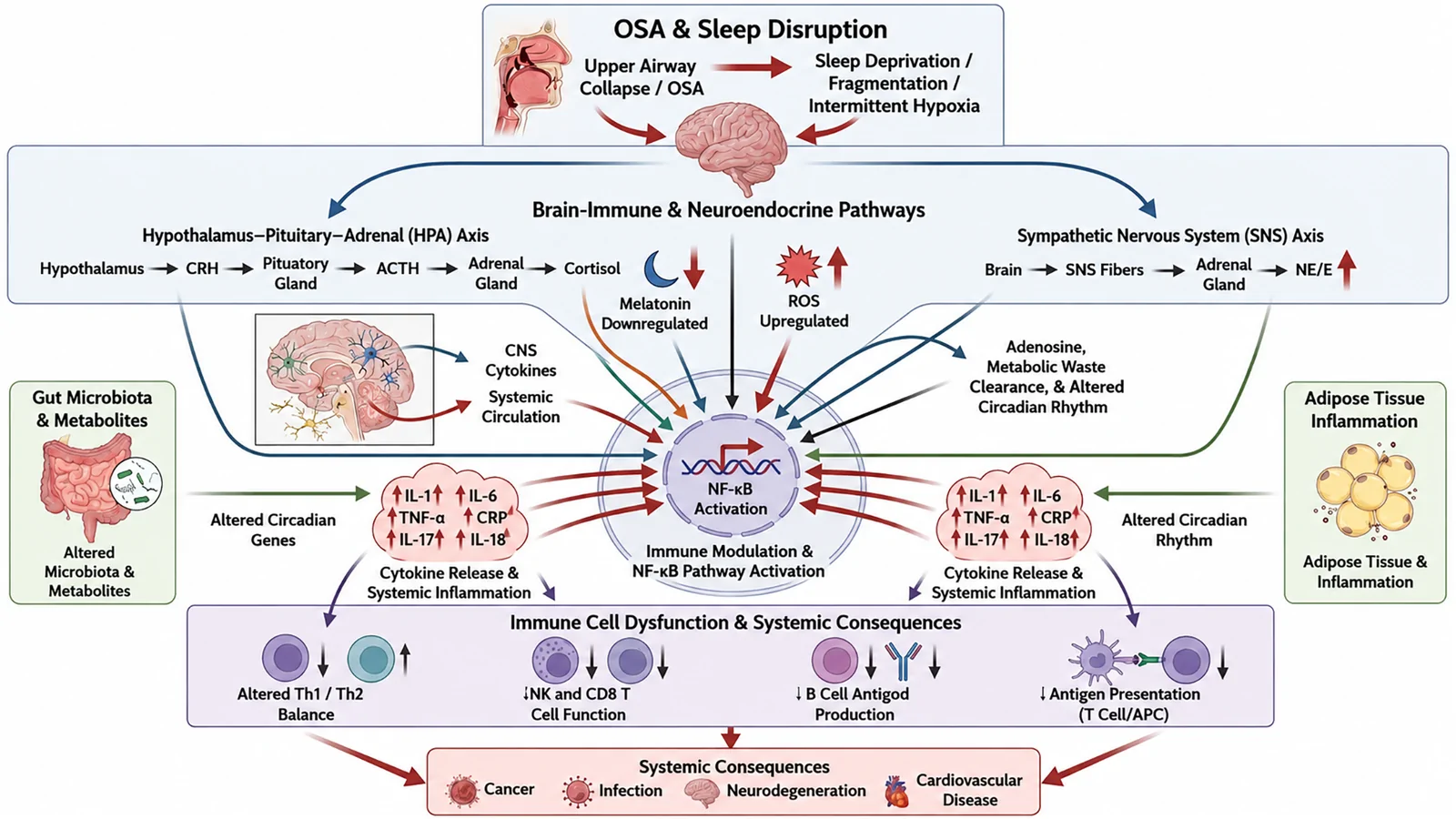
Proposed neuroimmune and inflammatory pathways linking obstructive sleep apnea to systemic immune dysregulation. Recurrent upper-airway obstruction in OSA produces intermittent hypoxia (IH), sleep fragmentation (SF), sympathetic activation, oxidative stress and circadian disruption, which may activate interconnected neuroendocrine and immune pathways. Altered hypothalamic–pituitary–adrenal (HPA) axis and sympathetic nervous system (SNS) activity may affect cortisol and catecholamine release, melatonin signaling, and reactive oxygen species (ROS) production. Changes in the gut microbiota and adipose-tissue inflammation may provide additional inflammatory signals. These processes can promote NF-κB activation and increased production of pro-inflammatory mediators including IL-1β, IL-6, IL-17A, TNF-α, CRP, and IL-18. Persistent inflammatory signaling may contribute to altered Th1/Th2 balance, NK- and CD8^+^ T-cell function, B-cell antibody production and antigen presentation. These immune alterations may result in OSA-associated cardiovascular, neurological, infectious and malignant complications. The figure presents proposed mechanistic relationships derived from clinical, animal and *in vitro* evidence; however, several pathways remain to be validated in human OSA cohorts. HPA, hypothalamus–pituitary–adrenal; CRH, corticotropin-releasing hormone; ACTH, adrenocorticotropic hormone; SNS, sympathetic nervous system; NE/E, norepinephrine/epinephrine; ROS, reactive oxygen species; CNS, central nervous system; NF-κB, nuclear factor kappa B; IL, interleukin; TNF-α, tumor necrosis factor-alpha; CRP, C-reactive protein; Th1, T helper 1; Th2, T helper 2; NK, natural killer; CD8, cluster of differentiation 8; APC, antigen-presenting cell.

Our review shows that evidence supporting an association between obstructive sleep apnea (OSA) and immune system dysfunction should be evaluated based on the degree of experimental support. Cross-sectional and longitudinal observational studies are consistent in documenting significant associations between elevated levels of circulating inflammatory cytokines such as IL-1β, IL-6, IL-8, IL-17, IL-18, TNF-α and CRP, and increased number of neutrophilic cells, monocytic cell subsets, T-cells, endothelial adhesion molecules and other immune related biomarkers. However, these studies primarily demonstrate association and none of the observational and cross-sectional studies can demonstrate that OSA is responsible for inducing these immunological aberrations (39, 60, 65, 69, 94, 101, 159, 174, 196, 232, 238, 240, 250, 282, 286, 287, 328). In addition to the association demonstrated by human observational studies, some human interventional studies demonstrate the contributory role of OSA, as continuous positive airway pressure (CPAP) and corrective surgery of upper airway obstructions were associated with decrease or partial normalization of selected inflammatory biomarkers and abnormal immune cell phenotypes. However, the extent and consistency of this response vary across different studies (118, 175, 267, 272, 284). In contrast to human observational and interventional data, animal studies and limited *in vitro* mechanistic studies on the effects of IH and SF are considerably stronger than human observational studies in providing evidence of direct biological causality. These studies provide the most direct experimental evidence that IH and SF can induce activation of inflammatory signalling pathways including those involving HIF-1α, NF-κB, NLRP3 and TLRs. The findings also show that IH and SF elicit pro-inflammatory macrophage/myeloid responses, disrupt the T helper 17/regulatory T-cell (Th17/Treg) balance and result in endothelial tissues inflammation (103, 108–110, 121, 126, 177, 180, 181, 189, 190, 208, 209, 244, 254–258, 270, 271, 291, 293). However, these *in vivo* and *in vitro* experimental findings support biological plausibility and causal mechanisms within controlled models, but their direct translation to human OSA requires caution. Thus, while human observational evidence strongly supports an association between OSA and immune dysregulation, interventional human studies provide more direct, although still heterogeneous, evidence of reversibility, whereas many specific molecular mechanisms remain predominantly supported by *in vivo* and *in vitro* models.

## 10 Conclusions and future directions

### 10.1 Conclusions

OSA is increasingly recognized as a systemic low-grade immunoinflammatory disorder. Its major hallmarks, including IH and SF, contribute to complex immune and inflammatory interactions. These responses may also be influenced by episodic hypercapnia and increased respiratory effort during sleep. The evidence summarized here indicates that OSA is associated with significant alterations in innate, adaptive and humoral immune responses.

Innate immune activation in OSA is characterized by increased pro-inflammatory cytokines and mediators, including IL-1β, IL-6, IL-8, TNF-α, CRP, MCP-1, ICAM-1, VCAM-1, HIF-1α, NF-κB, and inflammasome-related pathways. These changes vary considerably among patients, reflecting phenotypic heterogeneity. They contribute to oxidative stress, endothelial dysfunction, vascular inflammation, neural dysfunction and metabolic impairment. IH further activates monocytes, macrophages, neutrophils and endothelial inflammatory pathways, thereby sustaining chronic systemic inflammation.

Adaptive immune dysregulation is also a prominent feature of OSA. It includes enhanced Th1 and Th17 polarization, altered CD4^+^ and CD8^+^ T-cell responses, activation of PD-1/PD-L1 immune checkpoint signaling, and impaired regulatory T-cell (Treg) function associated with reduced FOXP3 expression. The resulting Th17/Treg imbalance appears to play a central role in sustaining chronic inflammation and endothelial injury. Alterations in NK, NKT and γδ T-cell populations further suggest impaired immune surveillance and disrupted cellular immunity. The evidence regarding humoral immune alterations remains limited. However, available studies indicate dysregulation of Th2-associated cytokines, complement pathways and anti-inflammatory cytokines, including IL-10, IL-12, IL-18, IL-35, and IL-37, which may contribute to both pro-inflammatory and compensatory immunoregulatory responses in OSA. Interpretation of these findings is complicated by the marked heterogeneity among studies, including differences in OSA severity, obesity status, age, comorbidities and methodological approaches. Nevertheless, the extant evidence indicates that OSA is not merely a sleep-related breathing disorder, but a condition characterized by extensive cross-talk between sleep disturbances, hypoxia-driven inflammation and innate, adaptive, and humoral immune dysregulation.

### 10.2 Future directions

Despite growing evidence linking OSA to immune and inflammatory dysregulation, several important knowledge gaps remain. Future studies should use standardized methods, including objective sleep assessment, consistent disease severity classification and controlled timing of biological sample collection. Variability in these factors may contribute to inconsistent findings across studies. Standardized multi-night sleep monitoring and time-controlled blood sampling may improve the reliability and comparability of immune and inflammatory biomarkers. Large, multicenter cohorts with rigorous BMI matching or adjustment and standardized sample-collection timing are needed to improve reproducibility. Independent validation cohorts should also be incorporated to establish the diagnostic and prognostic value of candidate immune biomarkers. Moreover, careful adjustment for major confounders—including sex, age, obesity, metabolic comorbidities, medication use, smoking status, and other coexisting conditions—is essential to distinguish OSA-specific immune alterations from inflammation attributable to associated clinical factors.

Further mechanistic investigations are needed to elucidate how IH and SF influence innate, adaptive and humoral immune pathways in OSA. Human studies specifically designed to distinguish the independent immune effects of intermittent hypoxia (IH) from those of sleep fragmentation (SF) are also needed. Particular attention should be given to inflammasome activation, Th17/Treg imbalance, PD-1/PD-L1 immune checkpoint signaling, complement activation and oxidative stress–related inflammatory pathways. The role of anti-inflammatory and compensatory immune responses, including those mediated by IL-10, IL-35 and IL-37, also remains incompletely understood. Additional longitudinal and interventional studies are required to determine whether immune dysregulation directly contributes to the cardiovascular, metabolic, neurocognitive and endothelial complications associated with OSA. Clinical interventional trials targeting gut microbiota through probiotics and specific inflammatory pathways should be prioritized to determine their therapeutic potential. Moreover, evidence regarding humoral immunity, autoimmunity, gut microbiota–immune interactions and immune cell functional alterations remain limited. Integrating molecular, cellular and clinical approaches in future translational studies may provide a more comprehensive understanding of immune–inflammatory cross-talk in OSA and facilitate the identification of novel biomarkers and therapeutic targets.
